# The Mosaic Genome of *Anaeromyxobacter dehalogenans* Strain 2CP-C Suggests an Aerobic Common Ancestor to the Delta-Proteobacteria

**DOI:** 10.1371/journal.pone.0002103

**Published:** 2008-05-07

**Authors:** Sara H. Thomas, Ryan D. Wagner, Adrian K. Arakaki, Jeffrey Skolnick, John R. Kirby, Lawrence J. Shimkets, Robert A. Sanford, Frank E. Löffler

**Affiliations:** 1 School of Civil and Environmental Engineering, Georgia Institute of Technology, Atlanta, Georgia, United States of America; 2 School of Biology, Georgia Institute of Technology, Atlanta, Georgia, United States of America; 3 Department of Microbiology, University of Iowa, Iowa City, Iowa, United States of America; 4 Department of Microbiology, University of Georgia, Athens, Georgia, United States of America; 5 Department of Geology, University of Illinois, Urbana, Illinois, United States of America; Centre for DNA Fingerprinting and Diagnostics, India

## Abstract

*Anaeromyxobacter dehalogenans* strain 2CP-C is a versaphilic delta-Proteobacterium distributed throughout many diverse soil and sediment environments. 16S rRNA gene phylogenetic analysis groups *A. dehalogenans* together with the myxobacteria, which have distinguishing characteristics including strictly aerobic metabolism, sporulation, fruiting body formation, and surface motility. Analysis of the 5.01 Mb strain 2CP-C genome substantiated that this organism is a myxobacterium but shares genotypic traits with the anaerobic majority of the delta-Proteobacteria (i.e., the *Desulfuromonadales*). Reflective of its respiratory versatility, strain 2CP-C possesses 68 genes coding for putative c-type cytochromes, including one gene with 40 heme binding motifs. Consistent with its relatedness to the myxobacteria, surface motility was observed in strain 2CP-C and multiple types of motility genes are present, including 28 genes for gliding, adventurous (A-) motility and 17 genes for type IV pilus-based motility (i.e., social (S-) motility) that all have homologs in *Myxococcus xanthus*. Although *A. dehalogenans* shares many metabolic traits with the anaerobic majority of the delta-Proteobacteria, strain 2CP-C grows under microaerophilic conditions and possesses detoxification systems for reactive oxygen species. Accordingly, two gene clusters coding for NADH dehydrogenase subunits and two cytochrome oxidase gene clusters in strain 2CP-C are similar to those in *M. xanthus.* Remarkably, strain 2CP-C possesses a third NADH dehydrogenase gene cluster and a cytochrome *cbb*_3_ oxidase gene cluster, apparently acquired through ancient horizontal gene transfer from a strictly anaerobic green sulfur bacterium. The mosaic nature of the *A. dehalogenans* strain 2CP-C genome suggests that the metabolically versatile, anaerobic members of the delta-Proteobacteria may have descended from aerobic ancestors with complex lifestyles.

## Introduction

Classification of the eubacterial domain remains a major challenge in prokaryotic taxonomy. Lateral gene transfer events introduce complexity that current classification methods rarely capture [1], [2]. 16S rRNA gene phylogeny is unreliable for predicting physiology but this analysis does typically provide information about an organism's evolutionary history [3], [4]. When applied to genomic analyses, phylogeny deduced from the 16S rRNA gene sequence provides a framework for using genomic information to interpret evolution by distinguishing derived traits from those of a common ancestor. *Anaeromyxobacter dehalogenans* strains were initially isolated from pristine soils based on their ability to derive energy from reductive dechlorination of chlorophenols [5], [6]. Characteristic for *A. dehalogenans* strains is great respiratory versatility including metal and radionuclide reduction and recent efforts have yielded additional isolates from contaminated subsurface environments and agricultural soils [7]–[10]. *Anaeromyxobacter* spp. are the first anaerobes that group with the order *Myxococcales* (traditionally called ‘myxobacteria’) according to 16S rRNA gene phylogeny.

Despite the dominance of anaerobes in the delta-Proteobacteria class, bacteria designated as myxobacteria have been unified as strict aerobes (reviewed in [11]). Myxobacteria are adapted to aerobic soil environments with changing nutrient availability. Myxobacteria form spores and fruiting bodies in response to unfavorable conditions, and use gliding motility and communal wolf pack behavior for predatory lifestyle [11], [12]. Many myxobacteria species are able to feed on and defend against other microorganisms using exoenzymes (e.g., proteases, nucleases, lipases, glucanases). Myxobacteria also produce secondary metabolites such as stigmatellin, saframycin, and myxovirescin with antifungal and antibacterial activities [13]. A common feature of myxobacteria is their extraordinary ability to sense and respond to complex environmental stimuli. For example, a multi-input signal transduction cascade tightly regulates fruiting body development and sporulation [14]–[16]. Additional characteristics that have been used to describe myxobacteria include large genome sizes around 10 Mb and high G+C contents in the range of 66–72% [11], [12]. Members of the *Myxococcales* include *Sorangium cellulosum*, *Stigmatella aurantiaca*, and the most extensively studied laboratory organism of this group, *Myxococcus xanthus*, which was the first to have a sequenced genome [17], [18]. Research on the nonpathogenic, free-living soil bacterium *M. xanthus* has led to the elucidation of many phenomena that were previously not known to exist in the prokaryotic domain such as coordinated social behavior, complex signal transduction networks, unique and complex motility mechanisms, and contact signaling [19]. Many of these complex and costly traits are lost in the absence of evolutionary pressure (e.g., following repeated transfers in rich medium) indicating their importance for survival in the soil environment [20]. Based on these unique observable traits, the myxobacteria were expected to constitute a distinct bacterial taxonomic domain [21]. When 16S rRNA gene classification placed the myxobacteria within the delta-Proteobacteria comprising bacteria whose primary distinction was anaerobic respiratory versatility rather than morphological and behavioral ingenuity, questions arose as to how such diversity originated within a coherent phylogenetic group (i.e., the delta-Proteobacteria) [22].

We used the genome sequence of *A. dehalogenans* strain 2CP-C (Accession number: CP000251) for comparative analysis with delta-Proteobacteria that share similar physiology (i.e., *Geobacteraceae*) and two phylogenetically closely related, aerobic myxobacteria. The genome analysis demonstrated that strain 2CP-C shares traits with strictly aerobic myxobacteria and anaerobic delta-Proteobacteria. The analysis provides evidence for ancient horizontal gene transfer from another bacterial domain and supports the hypothesis that respiratory versatility in *A. dehalogenans* is a derived trait, one that was gained after splitting from an aerobic ancestor that is common to the myxobacteria and possibly the entire delta-Proteobacteria class. We propose that the common ancestor of *M. xanthus* and *A. dehalogenans* was a facultative aerobe with an intermediate genome size of high G+C content that was capable of gliding motility, advanced signaling, sporulation, and flagellar motility.

## Results and Discussion

### Taxonomic Classification

**Figure 1:**
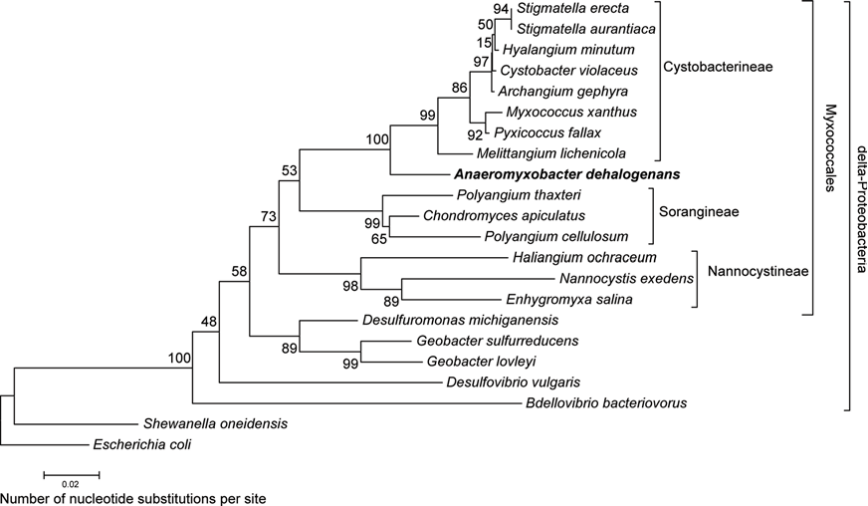
16S rRNA gene-based phylogenetic tree of the delta-Proteobacteria indicates that *A. dehalogenans* strain 2CP-C is deeply nested in the order Myxococcales. Neighbor-joining bootstrap values (500 replicates) are indicated at each branch. Class, order, and suborder designations are indicated on the right side.

**Figure 2:**
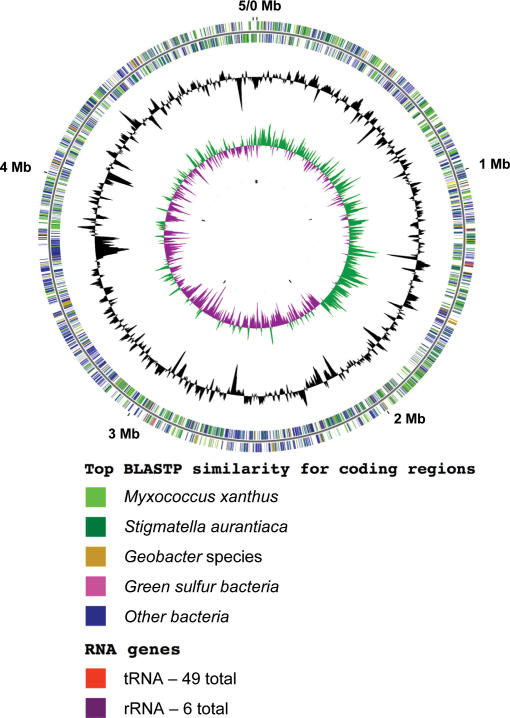
The *Anaeromyxobacter dehalogenans* strain 2CP-C complete genome with genes color-coded to indicate putative ancestry. Genome positions are indicated on the outside of the circle. The color legend refers to the two outer circles. The outer circle indicates predicted coding regions on the plus strand colored according to the organism that carries a gene with greatest sequence similarity. The second circle indicates predicted coding regions on the minus strand also color coded by gene similarity. The third circle (black) shows variation in G+C content with the average G+C value as the center line. The innermost circle (green and purple) depicts GC skew with the average GC skew as the center line.

The contributions of horizontal gene transfer (HGT) to bacterial evolution and speciation are currently unclear and estimates range from minimal to very relevant [2], [4]. Although the 16S rRNA gene is not immune from transfer between organisms [23], it is generally accepted that this gene is a phylogenetic marker that depicts evolutionary history in most cases [3]. According to 16S rRNA gene phylogeny, *A. dehalogenans* is a delta-Proteobacterium that is deeply nested in the order Myxococcales (Figure 1). Surprisingly, the *Anaeromyxobacter* suborder falls between the *Cystobacterineae* and the other two suborders, *Sorangineae* and *Nannocystineae*, implying that *A. dehalogenans* bears more relation to *M. xanthus* than the other two myxobacteria suborders (Figure 1). In accordance with this phylogenetic placement, 24.3% of genes on the strain 2CP-C genome have their highest top non-paralogous similarities (i.e., *E* values <10^−4^ based on the Bacterial Genome Subset, see Materials and Methods for details) to *M. xanthus* and 17.9% are most similar to genes in *Stigmatella aurantiaca* (Figure 2; Table S1). Only 6.4% of similar genes are shared between *A. dehalogenans* and the physiologically comparable delta-Proteobacterium *Geobacter sulfurreducens* (Figure 2; Table S1). About half of the total number of predicted genes had highest similarity to genes outside of the delta-Proteobacteria. No single organism outside the delta-Proteobacteria contributed more than 1.7% of the *Anaeromyxobacter* genome. For example, 1.7% of genes were similar to sequenced *Acidobacterium* genes, which make up an abundant but poorly understood phylum of acid-tolerant bacteria recently postulated to be a sister group to the delta-Proteobacteria [24]–[26]. Since the highest top non-paralogous similarity may not be statistically valid as the best estimate of closest relationship when there are several functional domains, 140 multi-domain proteins of particular functional interest were analyzed for the potential for multiple origins. Only three of the analyzed proteins (2.1%) demonstrated mixed origin. Our multi-domain sequence analysis suggests that genes of mixed origin do not occur frequently enough in the *A. dehalogenans* genome to affect the results of our large-scale analysis (Figure 2; Table S1).

**Figure 3:**
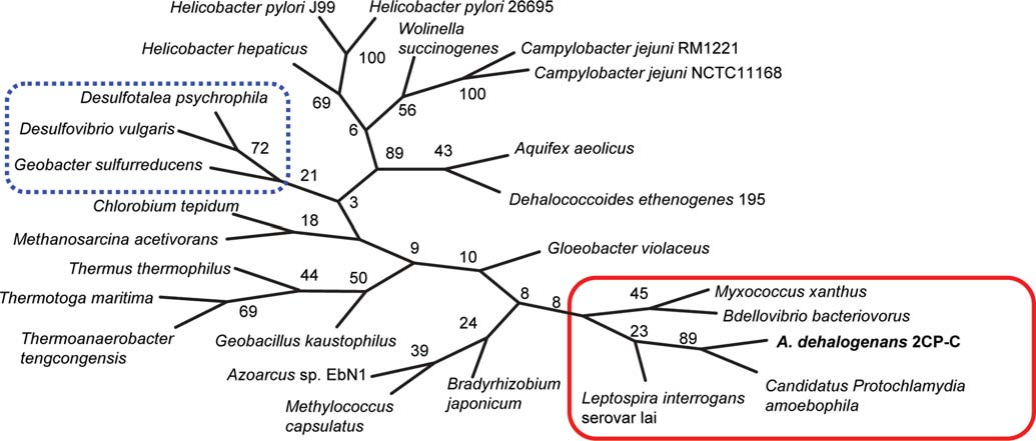
Phenetic, enzyme-based classification groups *A. dehalogenans* strain 2CP-C with aerobic organisms (red box). The blue dashed line box indicates the anaerobic delta-Proteobacteria included in the analysis. Bootstrap values are indicated at each node.

In order to use whole genome information to verify the remarkable evolutionary relationship between *A. dehalogenans* and the myxobacteria implied by 16S rRNA gene phylogeny, phenetic classification was performed based on the presence or absence of genes coding for enzymes with known function. Phenetic trees based on (putative) enzymatic capabilities have been used to classify organisms according to ecological niches occupied across all three domains of life [27]. This genome sequence-based classification divides the delta-Proteobacteria along the aerobic/anaerobic boundary and groups strain 2CP-C with the aerobes *M. xanthus* and *Bdellovibrio bacteriovorus* (Figure 3). The many genes associated with aerobic and predatory lifestyles present on the strain 2CP-C genome confirm the evolutionary relationship implied by 16S rRNA gene phylogeny and support classification of this organism as a member of the myxobacteria. Thus, phenetic classification puts forward the hypothesis that anaerobic metabolism is a derived trait in *A. dehalogenans*.

In order to test hypotheses regarding the evolutionary history in *A. dehalogenans,* we analyzed the strain 2CP-C genome for genes consistent with phylogeny as well as those consistent with physiology. Results indicate that the strain 2CP-C genome is made up of both, genes coding for known myxobacterial functions (i.e., predation, sporulation, motility, signaling) and genes that correspond to a versatile respiratory lifestyle.

### Features of the *A. dehalogenans* Strain 2CP-C Genome

The *A. dehalogenans* strain 2CP-C genome consists of a single, closed circular chromosome with 5,013,482 base pairs encoding 4,287 candidate protein-encoding genes (Table S1; Figure 2). The genome includes two copies each of the rRNA genes in two paralogous gene clusters and 49 tRNA genes distributed throughout the genome. The genome has a remarkably high G+C content of 74.9%, among the highest G+C percentage of any described organism. Organisms with similarly high G+C percentages, *Micrococcus luteus* (75%) and *Streptomyces griseus* (75%), are Gram-positive Actinobacteria. Among the Gram-negative bacteria, traditional myxobacteria are the only described organisms with high G+C content [11]. *M. xanthus* genomic DNA contains 68% G+C while the genomes of organisms in the suborders *Sorangineae* and *Cystobacterineae* range from 70-72% and 64-70%, respectively. Haywood-Farmer and Otto [28] recently presented a Brownian motion model to explain G+C content variation in closely related organisms, demonstrating that G+C content variation can be used to estimate the time lapsed since divergence from a common ancestor. Based on this model, G+C content suggests that *A. dehalogenans* is evolutionarily closer to the myxobacteria than to other delta-Proteobacteria families and that *A. dehalogenans* shares a common ancestor with the entire clade of myxobacteria, rather than representing an evolutionary intermediate (data not shown).

The higher than average G+C content facilitates the identification of recent gene acquisitions, as foreign DNA typically has lower G+C content. To begin HGT analysis, a total of 15 regions (including tRNA- and rRNA-coding regions) were identified having G+C contents below 70% (Figure 2, Table S2). Ten out of the 15 lower G+C regions contain genes with sequence similarities to phage- or transposon-related genes with *E* values of less than 0.01 (NCBI non-redundant database). No significant dinucleotide compositional difference was detected between low G+C regions and average G+C regions, indicating long residence time of horizontally-acquired sequences within the host genome [29]. However, rare codon usage in low G+C regions supports the hypothesis that these regions were horizontally transferred (Table S2). Based on G+C content and codon usage as indicators of recent HGT events, our analyses suggest that less than 0.2 Mb (4%) of the strain 2CP-C genome is attributed to HGT. These methods will likely fail to identify ancient HGT events because it is impossible to trace these genes' ancestral history, especially in cases where they were not maintained in other delta-Proteobacteria. Interestingly, gene locus Adeh_1877 has a BLASTP hit with 29% sequence identity to a *Myxococcus* phage Mx8 gene (*E* value = 9×10^−10^) suggesting that *A. dehalogenans* may be subject to infection by myxobacteria phages. In contrast to the limited HGT events in strain 2CP-C, HGT contributed at least 1.4 Mb of the 9.0 Mb (almost 16%) genome of *M. xanthus* (9). In addition to extensive HGT, frequent duplication events are manifested in the *M. xanthus* genome, which account for another 1.4 Mb [30]. The cause of the remaining 1.3 Mb genome size difference between *M. xanthus* and *A. dehalogenans* is documented in the GC skew of the strain 2CP-C genome.

In most eubacterial circular chromosomes, the excess of guanine relative to cytosine on the leading replicating strand (i.e., GC skew) aids in the detection of the origin and terminus of replication (23, 24). The origin and terminus are typically situated 180 degrees to each other on a circular chromosome [31]. However, the strain 2CP-C leading strand is about 1.5 Mb shorter than the lagging strand (Figure 2). This remarkable lack of symmetry may be a remnant of a large deletion event. An approximately 1.5 Mb contiguous region on the *M. xanthus* genome (position 4,300,000–5,800,000) codes for enzyme systems involved in secondary metabolism [30]. Secondary metabolite production, a signature feature of myxobacteria, is lacking in known *A. dehalogenans* strains and represents one of the major physiological differences between *A. dehalogenans* and traditional myxobacteria. The presence of secondary metabolite gene clusters in the common ancestor, and subsequent loss in *A. dehalogenans*, would explain the asymmetry between leading and lagging strand, as well as the genome size discrepancy with traditional myxobacteria.

These findings suggest that a hypothetical common ancestor to *M. xanthus* and *A. dehalogenans* had a genome of intermediate size that was both expanded by HGT and duplication events to 9.0 Mb in *M. xanthus* and trimmed, by one or more deletions, to 5.0 Mb in *A. dehalogenans*. The remaining 5.0 Mb of the strain 2CP-C genome reflect both the evolutionary history and respiratory innovation characteristic for the delta-Proteobacteria.

### Mosaic Nature of the Genome

Genes consistent with taxonomy together with those lacking counterparts in sequenced genomes of members of the Myxococcales order illustrate the mosaic nature of the strain 2CP-C genome. The sequence similarity of nearly half of the strain 2CP-C genes to myxobacterial genes roots *A. dehalogenans* in its taxonomic order while the foreign genes, which share highest similarities to genes from phylogenetically and physiologically diverse bacterial groups, elucidate the causes of functional diversity in this taxonomically coherent group.

#### Predation and Sporulation

Predation and sporulation are common features of previously characterized myxobacteria and these functions were used as defining traits for the taxon [32]. Unfortunately, the genes required for predation have been largely unexplored. Based on the knowledge available, the genes required for predation include *asgA*, *asgC*, *asgE*, *sdeK*, *csgA*, *frzABEFZ*, and the A motility system (specific genes tested include *aglB* and *cglB*) [33]. While predation has not been observed with *A. dehalogenans*, the *frz* genes and several A motility genes are present on the 2CP-C genome, and genes encoding chaperones and proteases implicated in predatory behavior in *M. xanthus* are present in multiple copies on the 2CP-C genome (Tables S3 and S4; [30]). Other predation genes including A- and C-signal genes were not found on the *A. dehalogenans* genome (e.g., *asgA, csgA*). Experimentally, predation has not been confirmed so it is unclear if *A. dehalogenans* is capable of a modified form of predation, if those genes have alternate functions, or if the genes are remnants of a formerly complete set of genes required for predatory lifestyle.

Like predation, sporulation has not been observed in *A. dehalogenans* under laboratory conditions. In contrast to the predation genes, of which the necessary complement is unknown, a known cadre of genes is required for sporulation in *M. xanthus.* Strain 2CP-C possesses putative gene homologs responsible for sporulation in *M. xanthus*, though essential genes for fruiting body development and sporulation in *M. xanthus* (e.g., *devRS* and *fruCD*) [34], [35] are lacking in strain 2CP-C. The presence of sporulation genes implies that *A. dehalogenans* once possessed the ability to sporulate but the paucity of genes remaining, and the lack of experimental evidence for sporulation, suggest that this behavior has been lost. Sporulation is a mechanism to survive unfavorable environmental conditions, which, in the case of the surface soil-dwelling myxobacteria, includes desiccation and rapidly changing nutritional conditions. Thus, differences in the genomes of two related organisms (i.e., presence versus absence of a complete set of sporulation genes) reflect a divergence in ecological niche speciation and survival strategy.

#### Surface Motility

**Figure 4:**
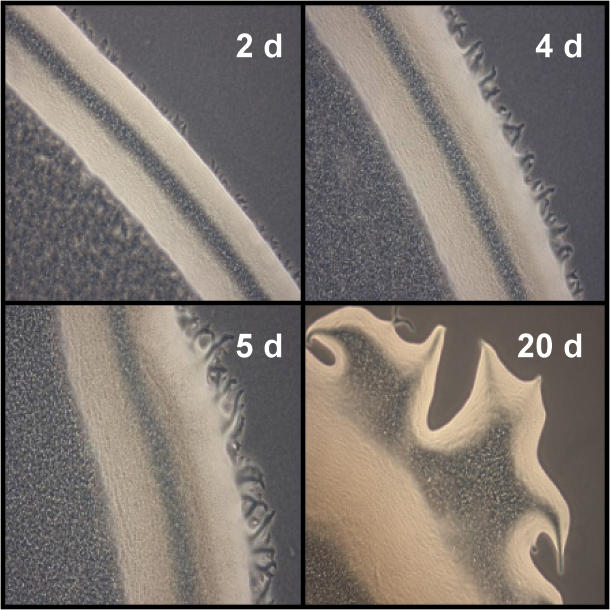
*A. dehalogenans* strain 2CP-C colony edges magnified 100-fold bear evidence of gliding motility. Flares characteristic of gliding motility began forming after 2 days of incubation.

Motility in strain 2CP-C occurs on solid surfaces (Figure 4), resembling the pattern of social motility observed in *M. xanthus* [36]. Many of the adventurous (A-) motility genes identified in *M. xanthus* are present in strain 2CP-C (Table S3) but whether or not these genes are sufficient for A-motility is unclear. Type IV pilus-based social (S-) motility, encoded by *pil* genes, is also partially responsible for *M. xanthus*' ability to move along surfaces (such as soil particles) [37]. Synteny and homology for the *pil* genes is shared between *M. xanthus* and strain 2CP-C (Figure S1A). Additionally, at least eight other genes implicated in social motility in *M. xanthus* have sequence similarity to genes on the strain 2CP-C genome. Interestingly, these genes are located in the region of the *M. xanthus* genome (position 4,300,000–5,800,000) that is absent in *A. dehalogenans* suggesting that gene rearrangement prior to the deletion event or gene acquisition following the deletion event have occurred. In any case, motility gene synteny and homology imply that *M. xanthus* and *A. dehalogenans* share a common ancestor that used type-IV pilus-based S-motility.

Within the delta-Proteobacteria, type IV pilus-based motility has only been observed in the myxobacteria, despite the presence of type IV pilus genes on the genomes of both *Bdellovibrio bacteriovorus* and *G. sulfurreducens* *[38]*, *[39]*. The *G. sulfurreducens* pilin encoded by *pilA* was implicated in biofilm formation and electron transfer to insoluble iron oxides outside of the cell but has not been shown to be involved in motility [40], [41]. *pil* gene synteny is shared between *M. xanthus,* strain 2CP-C, and *G. sulfurreducens* but sequence is not as well conserved in *G. sulfurreducens* (Figure S1A). For example, the 273 bp *pilA* gene in *G. sulfurreducens* is considerably smaller than *pilA* in *M. xanthus* (663 bp) or strain 2CP-C (711 bp). The *G. sulfurreducens pil* genes are divided into two clusters that lack several of the genes present in the myxobacteria clusters. However, some non-*pil* genes are conserved in the *pil* clusters in *M. xanthus*, strain 2CP-C and *G. sulfurreducens*. For example, the *accBC* genes are present in the *pil* clusters across genera suggesting that these genes play roles in pilus formation and function. The *accB* and *accC* genes are frequently located in a two-gene operon and regulated together to control biotin synthesis [42]. While a separate *accAB* cluster (Mxan_0081-0082) has been characterized in *M. xanthus* previously, the function of these two *acc* genes at the end of the pilus gene cluster (Mxan_5767-5768) are unclear [43]. Also conserved across all three genera is a *ribF* gene implicated in riboflavin biosynthesis that is located upstream of the *pil* cluster. The appearance of a conserved *ribF* gene upstream of a motility gene cluster in these three organisms reinforces the idea that riboflavin may have unexplored functions in the delta-Proteobacteria. There are no known interactions between *pil* genes and the *acc* or *rib* genes at this time. However, the presence of genes coding for type IV pili in all three organisms, arranged in syntenous clusters along with other conserved genes, suggests that the ancestor to the delta-Proteobacteria contained a similar set of type-IV pilus genes (and other genes) that may or may not have been involved in motility.

#### Signal Transduction

Like *M. xanthus*, strain 2CP-C possesses a large number of signal transduction genes to process information from its complex environment. Those organisms whose genomes contain multiple chemotaxis-like gene clusters have recently been shown to use chemosensory systems to regulate alternative functions. For example, transcription is regulated by one of the eight chemotaxis-like systems in *M. xanthus* [14], [44]. The multitude of chemosensory systems capable of responding to concentration gradients is thought to be required for temporal regulation of many aspects of physiology, including behavior [14].

Four of the seven chemotaxis gene clusters in strain 2CP-C share a high degree of synteny with *M. xanthus* clusters including *frz*, *dif*, *che*6, and *che*8 (Figure S2) [45]. The *dif* and *frz* clusters in *M. xanthus* are involved in extracellular polymeric substance (EPS) production, gliding motility, and fruiting body formation [46]–[48], and these gene clusters may play a similar role in *A. dehalogenans*. Strain 2CP-C possesses homologs to gliding motility genes *aglR* and *aglS* within the *dif* cluster, suggesting that the relationship between *agl* and *dif* genes may hold for *A. dehalogenans* as well as for *M. xanthus* (Figure S2). While spatial correlation between motility and regulatory genes on the genome has not been identified for *M. xanthus,* the strain 2CP-C genome provides more than one example of this. The *mglA* gene, which regulates both the adventurous and gliding motility systems in *M. xanthus* [49], [50], is associated with the *dif* cluster in strain 2CP-C. In addition, the *frz* cluster in strain 2CP-C is located upstream of genes coding for type IV pilus-based motility. A lack of proximity of genes with correlated functions in the *M. xanthus* genome in these two instances may be a result of the genome expansion that has taken place in *M. xanthus*. Thus, genome organization in strain 2CP-C assists in elucidating motility and regulatory pathways in *M. xanthus* by bringing to light possible protein interactions that have not been identified in the more complex *M. xanthus* genome.

#### Flagellar Motility

While swimming motility is common among the known delta-Proteobacteria, previously characterized myxobacteria do not display flagellar motility [51]. Three published myxobacterial genomes, each of which does not contain flagellar genes, reflect visual observations regarding motility [30], [52]. Surprisingly, the genome of strain 2CP-C includes a 55.7 kb cluster of genes coding for flagellar proteins including *motA, motB,* and *fliC* as well as more than 30 other genes with highest sequence similarities to genes implicated in flagellar motility [53] (Figure S1B). According to the currently accepted *Salmonella* and *Escherichia coli* models, almost all the genes necessary for flagellum synthesis and export are present in the strain 2CP-C cluster with the exception of the chaperone genes *fliJ*, *flgN*, *fliT*, and *flgA*, a rod capping protein *flgJ*, and a hook-length-control protein *fliK* (Table S5) [53]. Swimming motility has been observed in *A. dehalogenans* strain K cultures grown in liquid medium with nitrate as electron acceptor but this behavior is not commonly observed in strain 2CP-C. In order to determine whether flagellar motility in *A. dehalogenans* was retained from the delta-Proteobacterial ancestor or acquired after diversion from the myxobacteria, gene sequences and cluster synteny of strain 2CP-C were examined to document these genes' ancestry. The highest similarity to genes in the strain 2CP-C flagellar cluster ranges throughout the Proteobacteria and Firmicutes including similarities with genes found in *Thermotogales*, *Planctomycetes*, and *Aquificae*. Since strain 2CP-C is the first member of the Myxococcales that possesses genes for flagellar motility, no known gene homolog exists in traditional myxobacteria while eight out of the 33 genes coding for components of the flagellum apparatus have sequence similarities to genes found in members of the *Geobacteraceae* (Table S5). The closest gene order to that of the *A. dehalogenans* strain 2CP-C flagellar genes is in the flagellar gene cluster of *Desulfuromonas acetoxidans* (Figure S1B). Synteny and sequence similarity among flagellar genes of delta-Proteobacteria suggest that the flagellar gene cluster in *A. dehalogenans* strain 2CP-C is more ancient than the splitting of the myxobacteria from the *Desulfuromonadales*, and that this gene cluster was not acquired by HGT. Gliding motility and a shift to a lifestyle in unsaturated media (e.g., soil) may have triggered the loss of flagellar genes or re-allocation of the flagellar machinery to other function(s) (e.g., non-flagellar type III secretion) in most myxobacteria. Retention or acquisition of the flagellar gene cluster in strain 2CP-C along with the ability for surface motility reflects an adaptation to the environments *Anaeromyxobacter* spp. occupy, which include surface soils, saturated and unsaturated subsurface environments, and freshwater sediments such as those associated with rice fields and wetlands.

#### Versatile Energy Metabolism

**Figure 5:**
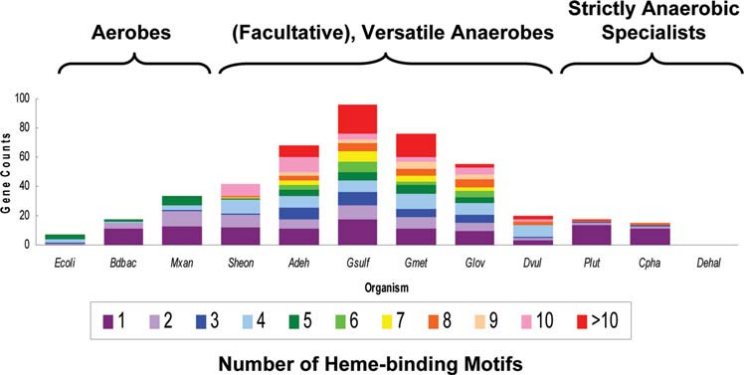
Distribution of heme-binding motifs in c-type cytochrome genes varies according to respiratory versatility and correlates with aerobic, facultative, or strict anaerobic lifestyle: aerobic bacteria, metal reducers, and obligate anaerobes. *Escherichia coli* (*Ecoli*), *Bdellovibrio bacteriovorus* (*Bdbac*), *Myxococcus xanthus* (*Mxan*), *Shewanella oneidensis* (*Sheon*), *Anaeromyxobacter dehalogenans* (*Adeh*), *Geobacter sulfurreducens* (*Gsulf*), *Geobacter metallireducens* (*Gmet*), *Geobacter lovleyi* (*Glov*), *Desulfovibrio vulgaris* (*Dvul*), *Pelodictyon luteolum* (*Plut*), *Chlorobium phaeobacteroides* (*Cpha*), and *Dehalococcoides* species strain BAV1 (*Dehal*).

Additional variations between the traditional Myxobacteria and *A. dehalogenans* manifest in the genes responsible for the remarkable respiratory versatility of *A. dehalogenans.* c-type cytochromes carrying one or multiple heme-binding sites are commonly involved in respiratory processes [54]–[56]. Accordingly, bacteria with low respiratory versatility possess a modest number of c-type cytochromes, which typically have fewer than five heme-binding motifs, whereas organisms with great respiratory versatility such as *Shewanella* spp., *Geobacter* spp., and *Anaeromyxobacter* spp. contain numerous c-type cytochrome genes, many of which have multiple heme binding motifs (Figure 5). The abundance of c-type cytochromes has consequences for the ecology of the host organism. *Shewanella* spp., *Geobacter* spp., and *Anaeromyxobacter* spp. occupy environments with variable redox conditions [57]–[61], whereas low numbers or the absence of c-type cytochromes limit an organism's environmental distribution. For example, *Dehalococcoides* strains lacking c-type cytochromes are restricted to low redox potential, anaerobic zones where reductive dechlorination is a feasible terminal electron accepting process [62]. The strain 2CP-C genome contains 68 putative c-type cytochrome genes with 57 containing multiple heme-binding motifs. In fact, the 40-heme cytochrome in strain 2CP-C has among the most putative heme binding sites of any described c-type cytochrome. Large numbers of hemes in c-type cytochromes may function as capacitors allowing organisms to store electrons during feast times to be used during famine and maintain the cell's metabolism under substrate-limiting conditions [56], [63], [64]. An analogous phenomenon is observed in eukaryotes where heme-containing ferritins store iron to outlast iron-shortages [65]. Strain 2CP-C possesses a gene for bacterioferritin, the prokaryotic corollary to ferritin [65], [66]. If large c-type cytochromes function as electron capacitors or if a varied collection of c-type cytochromes imparts the ability to respire across a broad redox spectrum (i.e., respiratory versatility), then large numbers of c-type cytochrome genes with multiple heme-binding sites may represent an evolutionary adaptation to life in environments with variable redox and substrate conditions.

Respiratory versatility is fueled by electrons derived from organic (e.g., acetate) or inorganic (e.g., hydrogen) electron donor oxidation. Strain 2CP-C possesses two Ni-Fe-type hydrogenases. One Ni-Fe hydrogenase large subunit gene (Adeh_0478) is highly similar in sequence to a gene in *G. sulfurreducens* (GSU0785) (*E* value of 0 and an amino acid similarity of 57%); this gene is included in the 1.7% of the genome most similar to the Acidobacterium *Solibacter usitatus* (*E* value of 0 and amino acid similarity of 64%). The other Ni-Fe hydrogenase large subunit gene (Adeh_4162) is an F420-reducing-type hydrogenase, which is located adjacent to a gene (Adeh_4163) coding for fused δ and γ Ni-Fe hydrogenase subunits. Adeh_4163 is one of three multi-domain proteins of mixed evolutionary origin that were found out of 140 multi-domain genes analyzed on the genome. The N-terminal domain of Adeh_4163 has its top non-*Anaeromyxobacter* blastp hit to FrhD, a methyl-viologen-reducing hydrogenase delta subunit in *Syntrophobacter fumaroxidans* MPOB (Sfum_1973; *E* value = 2e−34) [67] while the C-terminal domain aligns to an NADH ubiquinone oxidoreductase in *Candidatus Desulforudis audaxviator* MP104C (Accession number ACA60153; *E* value = 1e−62). Adeh_4162 is related to hydrogenase group 3 *vhuA* in strictly anaerobic, chlororespiring *Dehalococcoides* spp. (e.g., *Dehalococcoides* sp. strain BAV1 VhuA, *E* value of 3×10^−105^) [68]. These two types of hydrogenases may impart respiratory versatility under distinct environmental conditions (e.g., high versus low H_2_ partial pressures).

Similar to mixed-valence Ni-Fe clusters, iron-sulfur (Fe-S) clusters are commonly involved in electron transfer proteins. The strain 2CP-C genome codes for 42 different Fe-S domains whereas the *M. xanthus* genome (which encodes 34 c-type cytochromes) contains only 17 Fe-S domains. Apparently, abundances of c-type cytochromes and proteins with Fe-S domains correlate and convey respiratory versatility (Figure S3).

In accordance with its ability to perform respiratory reductive dechlorination (chlororespiration) [6], the strain 2CP-C genome contains two putative reductive dehalogenase (RDase) genes (Adeh_0329 and Adeh_0331), which have *E* values ranging from 2×10^−22^ (Adeh_0331) to 1×10^−12^ (Adeh_0329) compared to the *pceA* gene encoding the tetrachloroethene reductive dehalogenase of *D. hafniense* strain Y51 [69]. Each putative RDase contains an Fe_4_-S_4_ motif and a signal peptide characteristic for RDase genes (Figure S4, [70]). Distinguishing features of the putative RDases in strain 2CP-C are non-Tat signal peptides and internal transmembrane helices (Figure S4). In addition, while RDase genes are typically associated with an adjacent, downstream B gene encoding a small, hydrophobic protein with two or three transmembrane-spanning helices [71], the putative RDase genes Adeh_0329 and Adeh_0331 in the strain 2CP-C genome are associated with B genes that contain one and 10 transmembrane-spanning motifs, respectively. Linked with the strain 2CP-C RDase gene cluster is a gene (Adeh_0328) with five putative transmembrane-spanning motifs. Similar genes are associated with the putative tetrachloroethene RDase gene of *G. lovleyi* strain SZ and a putative RDase gene of *D. hafniense* strain DCB2; however, the genes of the latter two bacteria include an FMN binding domain, which is absent in strain 2CP-C (Figure S4) [72] . The function(s) of the transmembrane-spanning proteins including the RDase internal hydrophobic domains have not been explored, though it has been speculated that these hydrophobic regions are required for RDase functionality, possibly by anchoring the RDase to the membrane. Adeh_0331 is most similar to an uncharacterized RDase gene of *Desulfitobacterium hafniense* strain DCB-2 (*E* value of 2×10^−27^ and an amino acid similarity of 39%). While convergent evolution cannot be excluded, the high RDase gene similarity points towards horizontal gene transfer between a gram-negative delta-proteobacterium and a gram-positive bacterium. Interestingly, *A. dehalogenans* strain 2CP-C also possesses four genes encoding hydrolytic dehalogenases, one predicted haloalkane dehydrogenase (Adeh_0522) and three predicted haloacid dehalogenases (Adeh_0672, Adeh_3811, and Adeh_1218), suggesting that this organism's dehalogenation functions are not limited to reductive dechlorination.

### Oxygen Utilization and Detoxification

Due to its capacity for versatile anaerobic respiration, *A. dehalogenans* was hypothesized to bridge the evolutionary gap between delta-Proteobacteria with aerobic and anaerobic lifestyles [6]. However, *A. dehalogenans'* grouping within a subphylum inside the myxobacteria is inconsistent with this hypothesis (Figure 1). An alternative explanation is that the anaerobic versatile metabolism of *A. dehalogenans* is a result of convergent evolution arising from aerobic ancestry. The repertoire of respiratory genes and oxidative-stress-related genes in the strain 2CP-C genome includes genes characteristic for anaerobic and aerobic respiration, many of which have homologs in traditional myxobacteria (Figures S5, S6, S7, S8, Table S6). The analysis of genes involved in oxidative phosphorylation and defense against reactive oxygen species support the hypothesis that *A. dehalogenans* has an aerobic ancestor.

#### Oxidative Phosphorylation

In aerobic respiration, electron flow is initiated by an NADH dehydrogenase accepting electrons from an electron donor, and ends with cytochrome c oxidase, an enzyme system catalyzing the reduction of oxygen to water [73]. Fourteen genes encoding NADH dehydrogenase subunits in the strain 2CP-C genome produced closest BLAST hits to *M. xanthus* or *S. aurantiaca* homologs, and are located in two separate clusters, each of which is syntenous between all three organisms (Figure S5). Multiple sequence alignment of the first NADH dehydrogenase subunit (*nuoH*) suggest shared evolutionary history of this respiratory chain component between *A. dehalogenans*, *M. xanthus* and *S. aurantiaca* (Figure S5). Sequence similarities suggest further that many of the individual genes in these two myxobacterial NADH dehydrogenase gene clusters are homologous to those in the two large *G. sulfurreducens* NADH dehydrogenase gene clusters; however, the *G. sulfurreducens* clusters lack conserved gene order with the myxobacteria (gene order of both *G. sulfurreducens* clusters: *nuoABCDEFGHIJKLMN* compared to the separate *nuoEFJKLMN* and *nuoIHGDBA* clusters in the myxobacteria), suggesting more ancient divergence. Interestingly, the strain 2CP-C genome contains a third NADH dehydrogenase gene cluster located back-to-back with one of the conserved NADH dehydrogenase gene clusters characteristic for myxobacteria (Figure S5). This third cluster contains genes with sequence similarity and conserved synteny to *Chlorobium phaeobacteroides* and *Pelodictyon luteolum* (*nuoH E* values of 1×10^−71^ and 1×10^−70^, respectively), both of which are strictly anaerobic green-sulfur bacteria (Figures S5 and S6) [74]–[76]. The other sequenced myxobacteria genomes do not contain genes with homology to the genes in the green sulfur bacterial NADH dehydrogenase gene cluster. Thus, strain 2CP-C possesses a unique assemblage of NADH dehydrogenase subunits including clusters that resemble those of both strict anaerobes and aerobes.

A cluster of cytochrome c oxidase genes is present in all of the sequenced delta-Proteobacteria even though many of these organisms are considered strict anaerobes. This observation suggests that aerobic, possibly microaerophilic growth has not been recognized, or that these enzyme systems fulfill a different function such as detoxification of oxygen and reactive oxygen species (ROS) [77]. *A. dehalogenans* strain 2CP-C possesses three gene clusters that code for the 3–4 subunits of cytochrome oxidases. The first gene cluster encoding cytochrome oxidase subunits is homologous and syntenous to two cytochrome c oxidase clusters in *M. xanthus* and one in *S. aurantiaca* (Figures S7 and S8A). A second cytochrome oxidase gene cluster shares highest sequence similarity with the cytochrome c oxidase genes in the *Bdellovibrio bacteriovorus* genome but the gene synteny is most similar to the *M. xanthus* and *S. aurantiaca* gene clusters (Figures S7 and S8B). The third strain 2CP-C cytochrome oxidase gene cluster codes for a cytochrome *cbb*_3_ oxidase, a distinctive class of proton-pumping, respiratory heme-copper proteins reducing O_2_ to water [78]. Like the genes for the anaerobic NADH dehydrogenase, the cytochrome *cbb*_3_ oxidase genes are homologous and syntenous to genes in *Chlorobacteriaceae* (Figures S7 and S8C). These patterns of oxidative phosphorylation gene similarity imply that the myxobacteria have a common ancestor that reduced O_2_ to water as an energy-yielding respiratory process involving electron transfer to oxidized nicotinamide adenine dinucleotide (NAD^+^). Additionally, the oxidative phosphorylation gene clusters with sequence similarity to strict anaerobes explain the anaerobic respiratory versatility of *A. dehalogenans.* The synteny and sequence similarity among oxidative phosphorylation genes imply that the delta-Proteobacteria, including the distantly related *B. bacteriovorus,* share a common ancestor capable of aerobic respiration. Further, the presence of a green sulfur bacteria-like *cbb*_3_-type cytochrome oxidase gene cluster in *A. dehalogenans* strain 2CP-C that includes genes with sequence similarity to other anaerobic delta-Proteobacteria genes suggests that the respiratory versatility found in the extant delta-Proteobacteria is actually an innovation on aerobic respiration made possible by acquisition of foreign genes.

#### Defense Against Reactive Oxygen Species (ROS)

Oxidative phosphorylation generates ROS, for which aerobic organisms developed defense mechanisms to avoid detrimental effects [79]. It is not uncommon for catalase and superoxide dismutase to occur in organisms described as strict anaerobes to protect the cells from ROS [80]. While the *A. dehalogenans* strain 2CP-C genome does not contain genes for catalase, superoxide reductase, or cytochrome c peroxidases, genes encoding superoxide dismutases of the Mn and Fe type are present (Table S6). The genome also contains four rubrerythrin homologs. Rubrerythrin is a non-heme containing iron enzyme system that catalyzes the conversion of hydrogen peroxide to water in *D. vulgaris* [81]. *A. dehalogenans* also possesses two alkylhydroperoxidases, which are responsible for hydrogen peroxide conversion to water in *M. xanthus*. Hence, *A. dehalogenans* has combined aerobic and anaerobic strategies for ROS detoxification. Neelaredoxin and desulfoferrodoxin superoxide reductases, both non-heme containing iron enzyme systems that couple cytochrome c oxidation to superoxide reduction, are common ROS detoxifying enzymes in anaerobic organisms [82]–[84] but, of the delta-Proteobacteria surveyed, only *D. vulgaris* has superoxide reductase (Table S6). Interestingly, *G. sulfurreducens* seems to possess the full aerobe-type antioxidant enzymatic machinery, with genes encoding catalase, superoxide dismutase, and peroxidase in addition to the complete suite of genes required for oxidative phosphorylation and, yet, this organism is not able to grow with atmospheric oxygen concentrations. Many bacteria initially characterized as strict anaerobes, including *Geobacter sulfurreducens*, have subsequently been shown to consume oxygen at sub-atmospheric concentrations [84]–[86]. The presence of ROS detoxifying enzymes in the anaerobic delta-Proteobacteria support the hypothesis that the organisms classified as delta-Proteobacteria descended from an aerobic ancestor.

### Conclusions

The *A. dehalogenans* strain 2CP-C genome provides evidence that, contrary to the prevailing wisdom, aerobic organisms can be ancestral to anaerobes. While a duality is implied by the terms ‘aerobic’ versus ‘anaerobic’ environment, the soil rarely contains such definite distinctions. Soil-dwelling organisms are subject to frequently changing redox conditions, which govern their ecophysiology and consequently impact bioremediation practice [87], [88]. The delta-Proteobacteria, being primarily sediment- and soil-dwelling, are the only class of Proteobacteria that is dominated by anaerobes. A unique evolutionary history involving an aerobic ancestor may explain why all of the sequenced delta-Proteobacteria genomes contain genes encoding the cytochrome c oxidase complex, implicated in O_2_ reduction to water. *G. sulfurreducens*, a delta-Proteobacterium characterized by its anaerobic versatile lifestyle, has a genome that shares many characteristics with aerobes. The two recognized groups of aerobic delta-Proteobacteria are *B. bacteriovorus* and the myxobacteria, which are only distantly related to one another but show homology in genes coding for aerobic respiratory pathways. Because aerobic organisms do not form a monophyletic clade, it is widely accepted that aerobic metabolism arose several times independently in evolutionary history [77]. However, the distribution of aerobic organisms in all three myxobacterial suborders belies independent evolution of aerobic metabolism among the myxobacteria. These findings, along with the phylogeny and genome characteristics of *A. dehalogenans* strain 2CP-C, point to an alternate evolutionary history for this major bacterial class. Contrary to the hypothesis that anaerobic metabolism in the *Desulfuromonadales* or *Desulfovibrionales* was the ancestral metabolism from which myxobacterial aerobic respiration evolved, the detailed analysis of the *A. dehalogenans* strain 2CP-C genome suggests that in respiratory diversification within the delta-Proteobacteria class, it was the versaphilic anaerobes that innovated and the aerobic *Myxococcales* and *Bdellovibrionales* that were conservative.

## Materials and Methods

### Genome sequencing

Genomic DNA of *A. dehalogenans* strain 2CP-C was extracted from whole cells grown anoxically in non-reduced liquid R2A (Difco) complex medium without shaking at 35°C. DNA was extracted using the Qiagen genomic DNA extraction kit (Qiagen, Hilden, Germany). Genome sequencing was performed by the Department of Energy's Joint Genome Institute (JGI). The *A. dehalogenans* strain 2CP-C genome sequence has been assigned EMBL accession number CP000251.

### Gene prediction and annotation, phylogenetic and phenetic analyses

In addition to automated annotation provided by JGI, the high precision multi-genome scale annotation tool EFICAz was applied for refined annotation [89], [90]. Phylogenetic and molecular evolutionary analyses based on 16S rRNA gene sequences comparisons were conducted using MEGA version 3.1 [91]. ClustalW was used for multiple alignments and trees and bootstrap values were calculated using the Neighbor-Joining algorithm with default settings. Phenetic analysis based on enzymes was carried out as follows: first, 26 organisms with fully sequenced genomes were selected, including all available Proteobacteria from the delta and epsilon subdivisions, and representative species of other phyla. Enzyme function annotations for these organisms in the KEGG database [92] were complemented with predictions made by EFICAz [89], a highly precise approach for enzyme function inference that significantly increases annotation coverage [90]. Then, for each organism, a binary character table was constructed encoding the presence/absence of 1,272 different enzymes classified according to the Enzyme Commission system [93]. This character matrix was used as input for the SEQBOOT, DOLLOP and CONSENSE programs from the PHYLIP v3.66 phylogenetic package to generate a majority-rule consensus tree based on the Dollo parsimony method. DOLLOP and CONSENSE were run using default settings; the number of bootstrap samples generated by SEQBOOT was 100.

### Comparative genome analysis

A database of eubacterial protein sequences was compiled from the proteomes of fully sequenced bacterial genomes deposited in GenBank and the proteomes of draft bacterial genomes from JGI and The Institute for Genomic Research (TIGR) (Bacterial Genome Subset). Each *A. dehalogenans* strain 2CP-C protein sequence was then compared against this database using the BLAST software package [94] using a cutoff *E* value of 1×10^−5^ and default settings for all other parameters. Resulting BLAST output files were parsed using perl scripts to obtain top hits outside of the *A. dehalogenans* genome. Using the top non-paralogous hits, each coding sequence (CDS) in the circular chromosome was colored according to closest phylogenetic similarity.

### Survey of relevant genes having more than one functional domain

To detect putative chimeric genes coding for multi-domain proteins in strain 2CP-C, 140 amino acid sequences of proteins related to respiration, flagella assembly, pilus assembly, and signal-transduction were blasted against the NCBI non-redundant database at using default parameters and the conserved domains search option. Alignments of top blastp hits outside of *Anaeromyxobacter* spp. were viewed in the graphical output of NCBI blast and checked for correspondence to domains marked in the NCBI conserved domains output.

### Identification of HGT regions

Analysis of the *A. dehalogenans* strain 2CP-C genome for evidence of gene acquisitions via HGT was based upon base composition and codon usage patterns [95], [96]. Regions having a G+C content between the minimum of 57.5% and 70% were located on genome plots in Artemis and Cgview [97], [98]. Duplicated ribosomal RNA loci with G+C contents of 57.5% were excluded from further analysis. Artemis was used to manually select protein-coding genes outside the low G+C regions for codon counts and plot the Karlin signature, which compares local dinucleotide composition within a sliding window relative to dinucleotide composition of the entire genome [99]. Codon Adaptation Indices (CAI) for all protein-coding loci were computed with JCat using default parameters (www.jcat.de)[100]. The CAI measures codon usage deviation from average codon usage of the entire genome [101]. Occurrences of the codons TTA_Leucine_ and ATA_Isoleucine_ found in the strain 2CP-C genome only 52 times and 140 times, respectively, were manually tallied in Artemis. To determine the level of amino acid sequence similarity of translated gene sequences within and adjacent to low G+C regions, protein sequences were queried against a collection of phage proteomes and translated phage-related genes present in complete bacterial genomes from NCBI and IMG. Genes from low G+C regions not containing phage gene BLAST hits were then compared against the NCBI non-redundant database to confirm the absence of horizontally transferred sequences. The BLAST *E* value provides an estimate of the probability that the similarity of a random hit of a query sequence to a hit [94]. Two regions located between Adeh_1913 and Adeh_1963 with 69.5% G+C content contain ribosomal proteins, show no deviation in codon adaptation, and were excluded from further analysis.

### Motility assays

*A. dehalogenans* was grown as described [6] and cells were harvested in mid-log phase. Cell suspensions (10 µL, ∼10^6^ cells) were transferred onto R2A solid medium (1.5% agar), allowed to dry, and incubated aerobically at 32°C.

### Microscopy

Surface motility was observed using Nikon SMZ10000 dissecting and Nikon Eclipse E400 phase-contrast microscopes. Images were captured with a digital camera and manipulated using QImaging software.

## Supporting Information

Figure S1 Gene orders of motility gene clusters of *A. dehalogenans* strain 2CP-C suggest diverse ancestry. Locus ID tags for the gene clusters are given in parentheses. Color-coding of bars indicates clusters with similar gene order. White, unlabelled genes/gene clusters are not conserved between the organisms. (A) Type IV pilus-based motility clusters of *A. dehalogenans* strain 2CP-C are syntenous with *M. xanthus*, and *G. sulfurreducens*. The *G. sulfurreducens pil* genes are divided into two clusters that are missing several of the genes present in the myxobacteria clusters. Conserved non-*pil* genes include *accBC* and *ribF*. (B) Flagellar motility clusters of *A. dehalogenans* strain 2CP-C and *Desulfuromonas acetoxidans* have conserved gene order. Embedded in the center of the *A. dehalogenans* flagellar cluster, between *motAB* and a cluster of *fli* genes, is a cluster of chemotaxis genes including two *cheA* genes, four *mcp* genes, and two response regulator *cheY* genes, whereas in *Desulfuromonas acetoxidans*, the complete flagellar gene cluster is downstream of a single chemotaxis gene cluster. Chemotaxis gene cluster bars are color-coded according to the genes present (see Figure S2).
(3.44 MB TIF)

Figure S2 Four chemotaxis gene clusters in *A. dehalogenans* strain 2CP-C are highly syntenous with *M. xanthus* clusters. Two clusters also show conserved gene order with *G. sulfurreducens*. Locus tag designations are either as indicated in public databases (IMG or NCBI) or interpolated from adjacent loci (i.e., *M. xanthus frz* and *dif* clusters). Arrows represent individual genes. Non-*che* genes are indicated in white. (A) The *M. xanthus frz* gene cluster is conserved in strain 2CP-C but not in *G. sulfurreducens*. (B) The *M. xanthus dif* gene cluster is conserved in both strain 2CP-C and *G. sulfurreducens*. (C) The *M. xanthus che*6 gene cluster is conserved in strain 2CP-C but not in *G. sulfurreducens*. (D) The *M. xanthus che*8 gene cluster is conserved in both strain 2CP-C and *G. sulfurreducens*.
(3.83 MB TIF)

Figure S3 A correlation exists between the number of genes containing Fe-S cluster motifs and the number of genes containing heme binding motifs for selected aerobic and anaerobic organisms. Outliers not included in the regression analysis are shown in red, open symbols. *Escherichia coli* (Ecoli), *Myxococcus xanthus* (Mxan), *Shewanella oneidensis* (Sheon), *Anaeromyxobacter dehalogenans* (Adeh), *Geobacter sulfurreducens* (Gsulf), *Geobacter metallireducens* (Gmet), *Geobacter lovleyi* (Glov), *Pelobacter carbinolicus* (Pcar), and *Dehalococcoides* sp. strain BAV1 (BAV1).
(2.11 MB TIF)

Figure S4 Gene order and domain structure of putative reductive dehalogenase gene clusters in *A. dehalogenans* strain 2CP-C are unique when compared to other putative reductive dehalogenase gene clusters. Locus tag designations are given in parentheses. Selected domains (determined by SMART [101] and http://www.cbs.dtu.dk/services/TMHMM-2.0) are indicated according to the legend. Arrows represent individual genes.
(3.32 MB TIF)

Figure S5 Gene order of NADH dehydrogenase gene clusters in *A. dehalogenans* strain 2CP-C indicates both phylogenetically consistent and foreign ancestry, representing aerobic and anaerobic organisms, respectively. Color-coding of arrows indicates clusters with similar gene order and genes with sequence similarity. (A) One group of myxobacteria-like NADH dehydrogenase (*nuo*, ubiquinone oxidoreductase) subunit genes is split into two separate clusters on the *A. dehalogenans* strain 2CP-C genome but its gene sequences are conserved among myxobacteria. (B) Two NADH dehydrogenase (*nuo*) gene clusters are located back-to-back on the *A. dehalogenans* strain 2PC-C genome. One of the coupled NADH dehydrogenase gene clusters is myxobacteria-like while the other has conserved sequence and gene order with green sulfur bacteria.
(3.08 MB TIF)

Figure S6 Multiple sequence alignment of *A. dehalogenans* strain 2CP-C NADH dehydrogenase subunit 1 genes (*nuoH*) indicates aerobic and anaerobic ancestry. Alignment was made with full-length genes (NCBI database). Locus ID tags for select organisms are indicated in parentheses.
(2.87 MB TIF)

Figure S7 Multiple sequence alignment of *A. dehalogenans* strain 2CP-C cytochrome oxidase subunit I genes (*ctaD* or *fixN*) indicates aerobic and anaerobic ancestry. Alignment was made with full-length genes from the NCBI database. Locus ID tags for select organisms are indicated.
(3.91 MB TIF)

Figure S8 Gene order of cytochrome oxidase gene clusters of *A. dehalogenans* strain 2CP-C indicate diverse ancestry. Color-coding of arrows indicates clusters with similar gene order and genes with sequence similarity. Colors represent gene annotations as follows: teal, Fe-S binding motif-containing genes; green, polysulphide reductase genes (*nrfD*); dark blue, synthesis of cytochrome c oxidase genes (*sco1*); light blue, cytochrome c oxidase subunit genes; pink, c-type cytochrome genes; yellow, *sox* genes; red-cytochrome *cbb3* oxidase subunit 1 genes (*fixN*); solid grey, cytochrome *cbb3* oxidase mono-heme subunit genes (*fixO*); striped grey, copper-translocating P-type ATPase genes; Orange, cytochrome *cbb3* oxidase maturation genes. Hypothetical or non-conserved genes are indicated in white. Locus ID tags are given in parentheses. (A and B) Cytochrome c oxidase clusters syntenous with aerobic organisms. (C) Cytochrome cbb3 oxidase clusters syntenous with anaerobic organisms.
(3.05 MB TIF)

Table S1 *Anaeromyxobacter dehalogenans* strain 2CP-C genome summary. Values indicated are based on both analyses from this study as well as automated annotation reflected in the NCBI genome database.
(0.04 MB DOC)

Table S2 Putative Horizontal Gene Transfer (HGT) regions based on deviating G+C content and Minimum Codon Adaptation Index (MCAI) HGT calculation was based on phylogenetic origin of lower G+C regions and regions of low codon adaptation index in the *A. dehalogenans* strain 2CP-C genome. Ten out of 15 G+C regions below 70% contain genes with sequence similarities to phage- or transposon-related genes with *E* values less than 0.01. Four additional putative HGT regions were identified by codon adaptation index. The genome average MCAI is 0.729.
(0.06 MB DOC)

Table S3 Genes for adventurous motility proteins on the *A. dehalogenans* strain 2CP-C genome imply that this type of motility is present. *E* values and identities given refer to *M. xanthus* sequences. Many of the gliding motility genes identified in *M. xanthus* are present in *A. dehalogenans* strain 2CP-C but whether or not the genes present are sufficient to produce gliding motility is unknown.
(0.05 MB DOC)

Table S4 The *A. dehalogenans* strain 2CP-C genome contains multiple copies of protease and chaperone genes. The *E* values and identities refer to a comparison with *M. xanthus* genes. Of the 19 genes identified encoding proteolytic enyzmes in the strain 2CP-C genome, 14 have homologs in *M. xanthus*.
(0.04 MB DOC)

Table S5 Flagellar motility genes on the *A. dehalogenans* strain 2CP-C genome. According to the currently accepted *Salmonella* and *E. coli* models, almost all the genes necessary for flagellum synthesis and export are present in a coherent cluster on the *A. dehalogenans* genome with the exception of four chaperone genes (*fliJ, flgN, fliT, flgA*), a rod capping protein *flgJ*, and a hook-length-control protein *fliK*.
(0.05 MB DOC)

Table S6 Reactive Oxygen Species (ROS)-detoxification gene comparison across selected delta-proteobacteria genomes (*Myxococcus xanthus* DK1622, *Anaeromyxobacter dehalogenans* 2CP-C, *Geobacter sulfurreducens* PCA, and *Desulfovibrio vulgaris* Hildenborough) indicates that the *A. dehalogenans* strain 2CP-C genome combines aerobic with anaerobic strategies for detoxifying ROS.
(0.04 MB DOC)
