# Supplementary figures and images for: The Mosaic Genome of Anaeromyxobacter dehalogenans Strain 2CP-C Suggests an Aerobic Common Ancestor to the Delta-Proteobacteria
Source: PLoS One. 2008 May 7;3(5):e2103. doi: 10.1371/journal.pone.0002103 (PMC2330069; doi:10.1371/journal.pone.0002103)

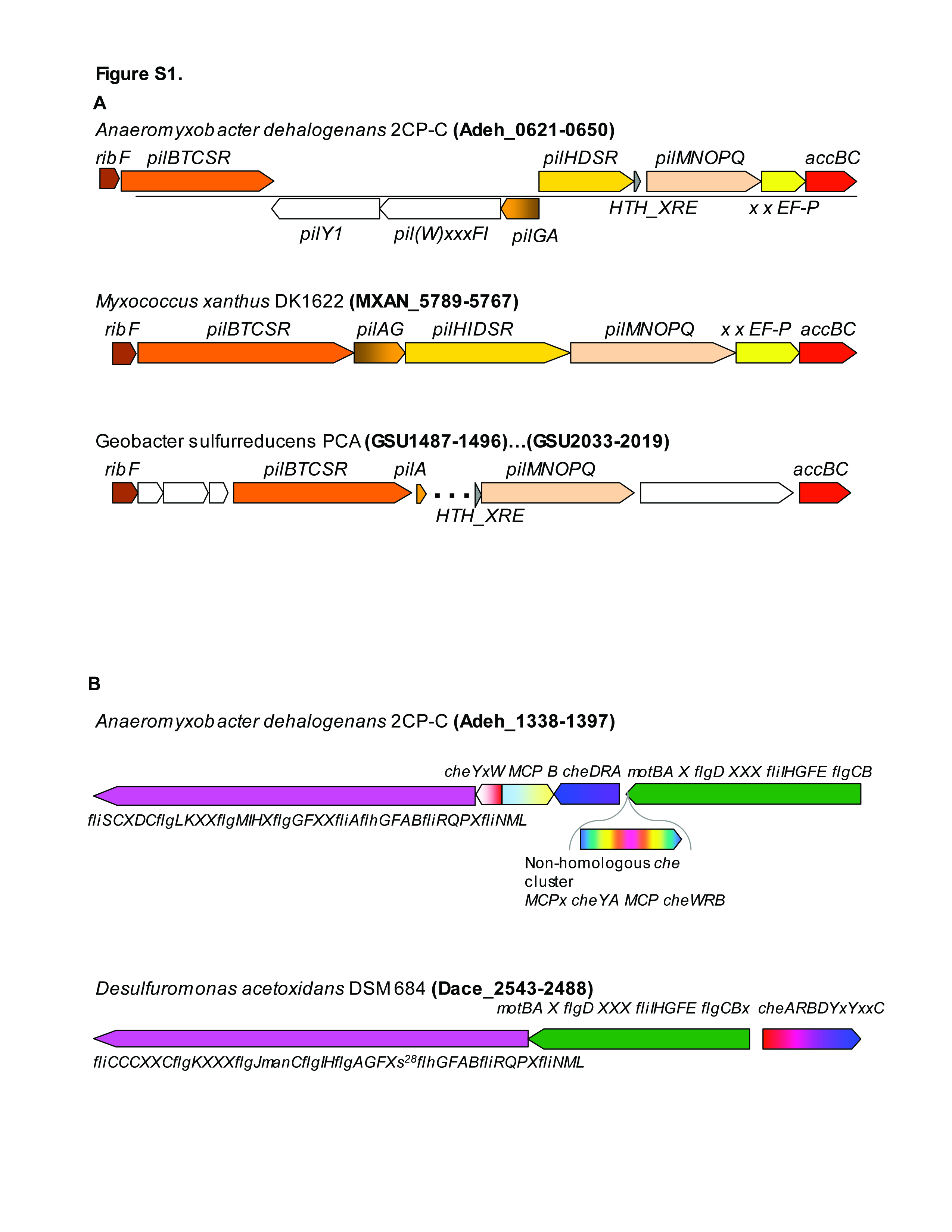

Supplement: Figure S1 — Gene orders of motility gene clusters of A. dehalogenans strain 2CP-C suggest diverse ancestry. Locus ID tags for the gene clusters are given in parentheses. Color-coding of bars indicates clusters with similar gene order. White, unlabelled genes/gene clusters are not conserved between the organisms. (A) Type IV pilus-based motility clusters of A. dehalogenans strain 2CP-C are syntenous with M. xanthus, and G. sulfurreducens. The G. sulfurreducens pil genes are divided into two clusters that are missing several of the genes present in the myxobacteria clusters. Conserved non-pil genes include accBC and ribF. (B) Flagellar motility clusters of A. dehalogenans strain 2CP-C and Desulfuromonas acetoxidans have conserved gene order. Embedded in the center of the A. dehalogenans flagellar cluster, between motAB and a cluster of fli genes, is a cluster of chemotaxis genes including two cheA genes, four mcp genes, and two response regulator cheY genes, whereas in Desulfuromonas acetoxidans, the complete flagellar gene cluster is downstream of a single chemotaxis gene cluster. Chemotaxis gene cluster bars are color-coded according to the genes present (see Figure S2). (3.44 MB TIF) [file pone.0002103.s001.tif]

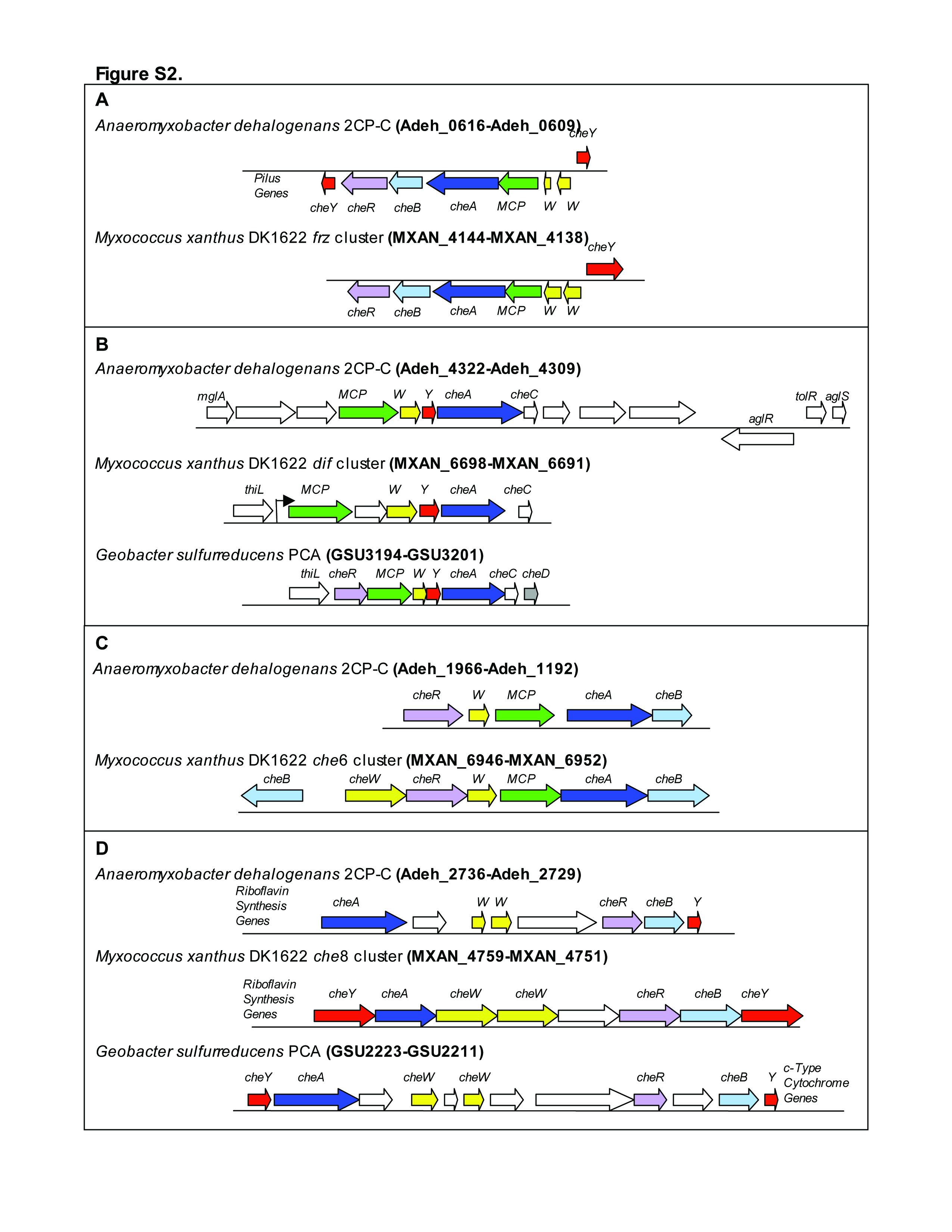

Supplement: Figure S2 — Four chemotaxis gene clusters in A. dehalogenans strain 2CP-C are highly syntenous with M. xanthus clusters. Two clusters also show conserved gene order with G. sulfurreducens. Locus tag designations are either as indicated in public databases (IMG or NCBI) or interpolated from adjacent loci (i.e., M. xanthus frz and dif clusters). Arrows represent individual genes. Non-che genes are indicated in white. (A) The M. xanthus frz gene cluster is conserved in strain 2CP-C but not in G. sulfurreducens. (B) The M. xanthus dif gene cluster is conserved in both strain 2CP-C and G. sulfurreducens. (C) The M. xanthus che6 gene cluster is conserved in strain 2CP-C but not in G. sulfurreducens. (D) The M. xanthus che8 gene cluster is conserved in both strain 2CP-C and G. sulfurreducens. (3.83 MB TIF) [file pone.0002103.s002.tif]

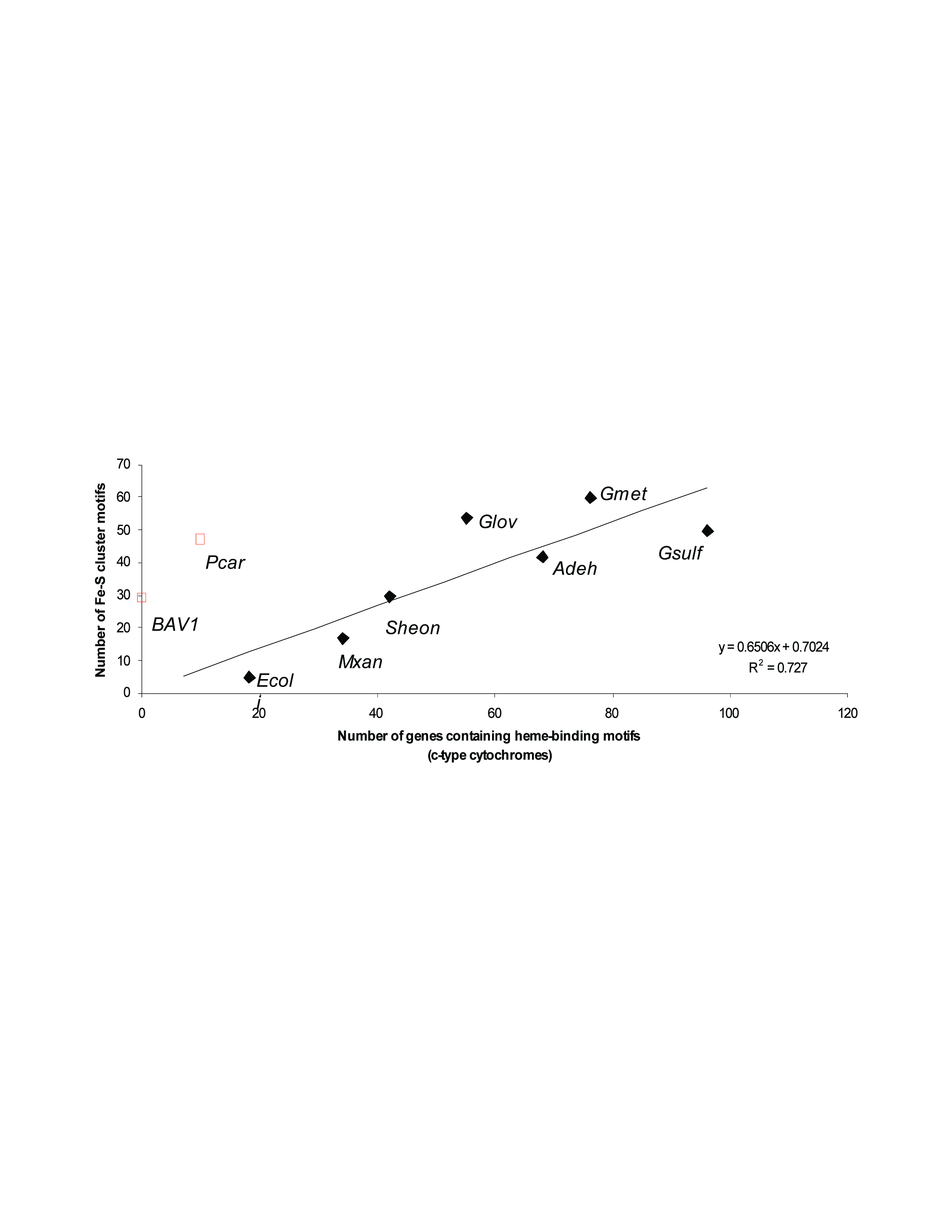

Supplement: Figure S3 — A correlation exists between the number of genes containing Fe-S cluster motifs and the number of genes containing heme binding motifs for selected aerobic and anaerobic organisms. Outliers not included in the regression analysis are shown in red, open symbols. Escherichia coli (Ecoli), Myxococcus xanthus (Mxan), Shewanella oneidensis (Sheon), Anaeromyxobacter dehalogenans (Adeh), Geobacter sulfurreducens (Gsulf), Geobacter metallireducens (Gmet), Geobacter lovleyi (Glov), Pelobacter carbinolicus (Pcar), and Dehalococcoides sp. strain BAV1 (BAV1). (2.11 MB TIF) [file pone.0002103.s003.tif]

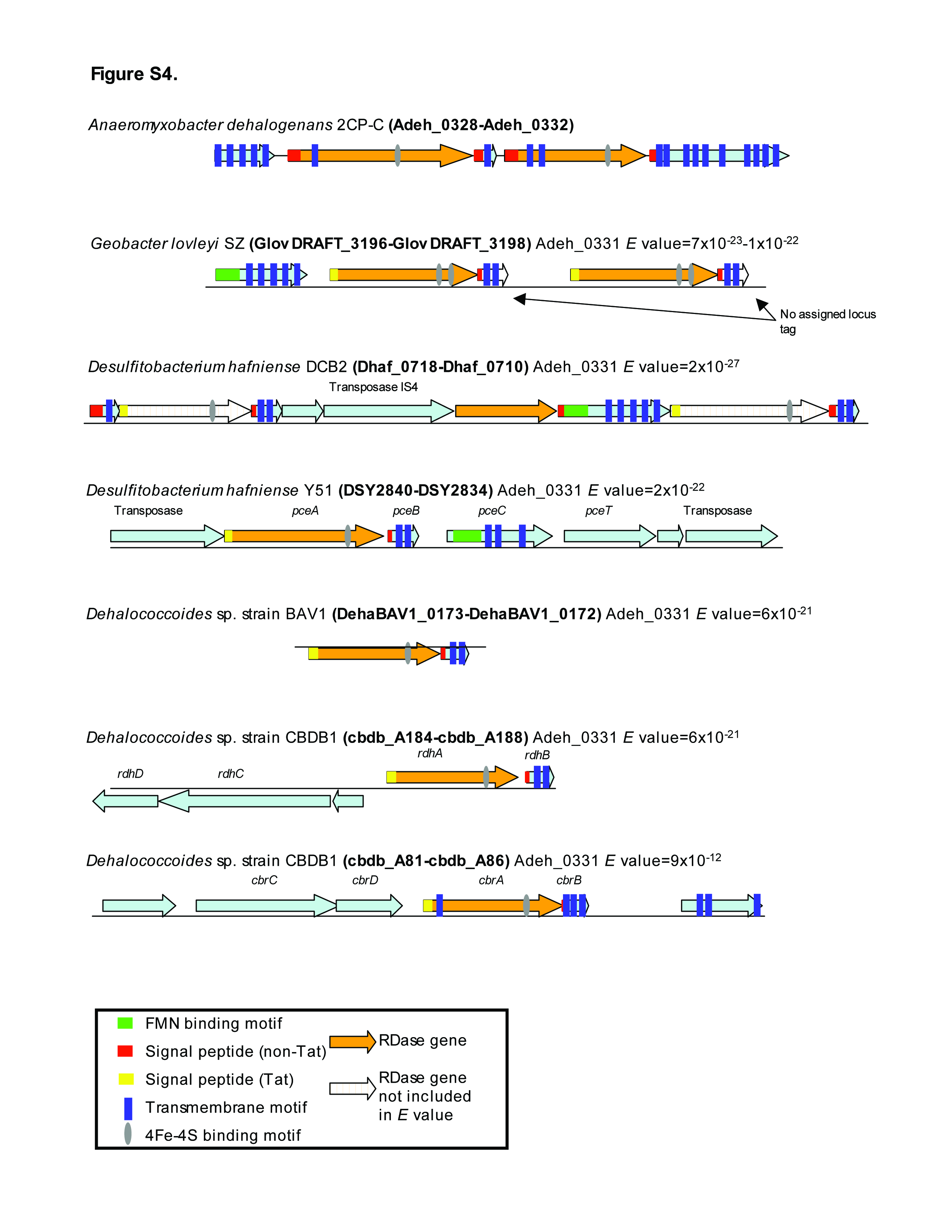

Supplement: Figure S4 — Gene order and domain structure of putative reductive dehalogenase gene clusters in A. dehalogenans strain 2CP-C are unique when compared to other putative reductive dehalogenase gene clusters. Locus tag designations are given in parentheses. Selected domains (determined by SMART [101] and http://www.cbs.dtu.dk/services/TMHMM-2.0) are indicated according to the legend. Arrows represent individual genes. (3.32 MB TIF) [file pone.0002103.s004.tif]

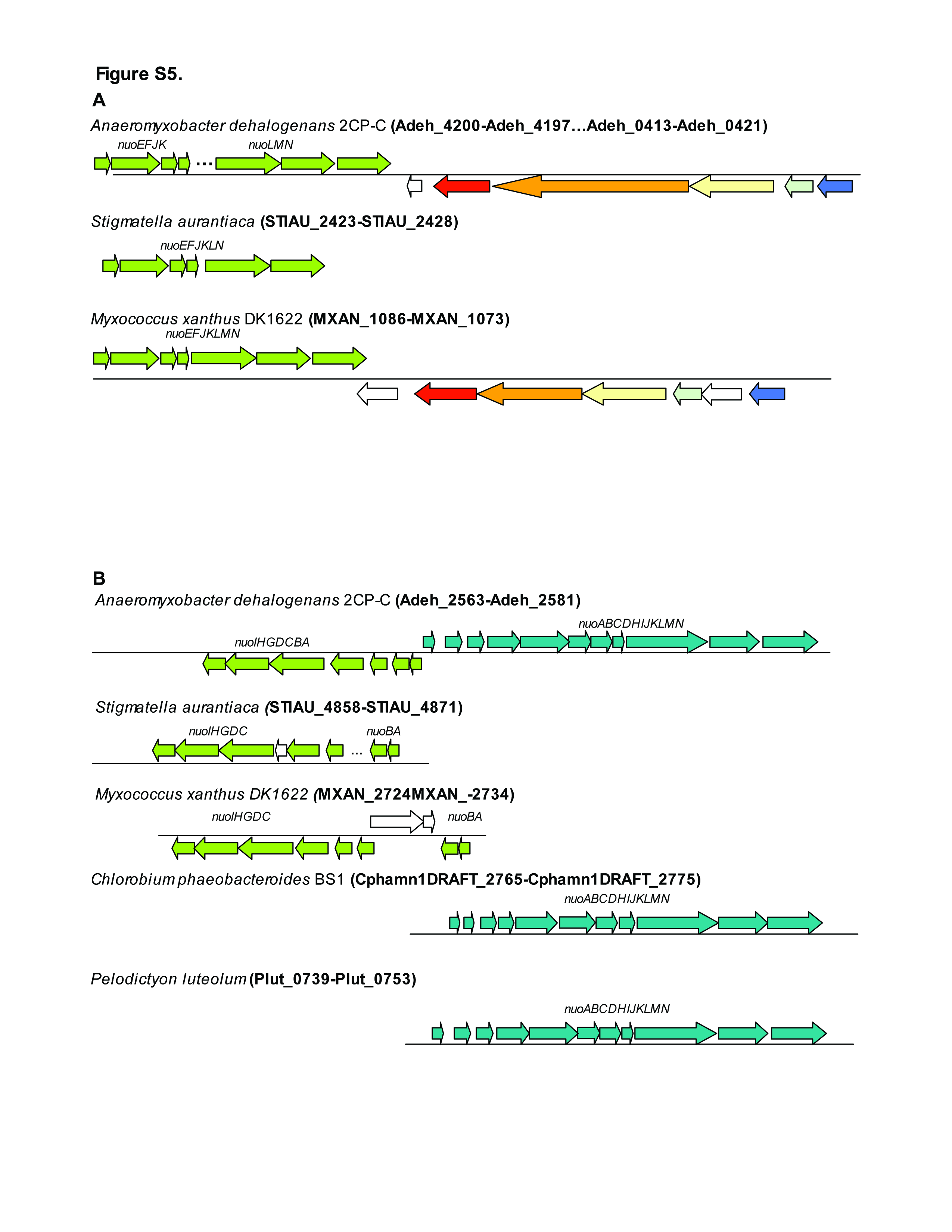

Supplement: Figure S5 — Gene order of NADH dehydrogenase gene clusters in A. dehalogenans strain 2CP-C indicates both phylogenetically consistent and foreign ancestry, representing aerobic and anaerobic organisms, respectively. Color-coding of arrows indicates clusters with similar gene order and genes with sequence similarity. (A) One group of myxobacteria-like NADH dehydrogenase (nuo, ubiquinone oxidoreductase) subunit genes is split into two separate clusters on the A. dehalogenans strain 2CP-C genome but its gene sequences are conserved among myxobacteria. (B) Two NADH dehydrogenase (nuo) gene clusters are located back-to-back on the A. dehalogenans strain 2PC-C genome. One of the coupled NADH dehydrogenase gene clusters is myxobacteria-like while the other has conserved sequence and gene order with green sulfur bacteria. (3.08 MB TIF) [file pone.0002103.s005.tif]

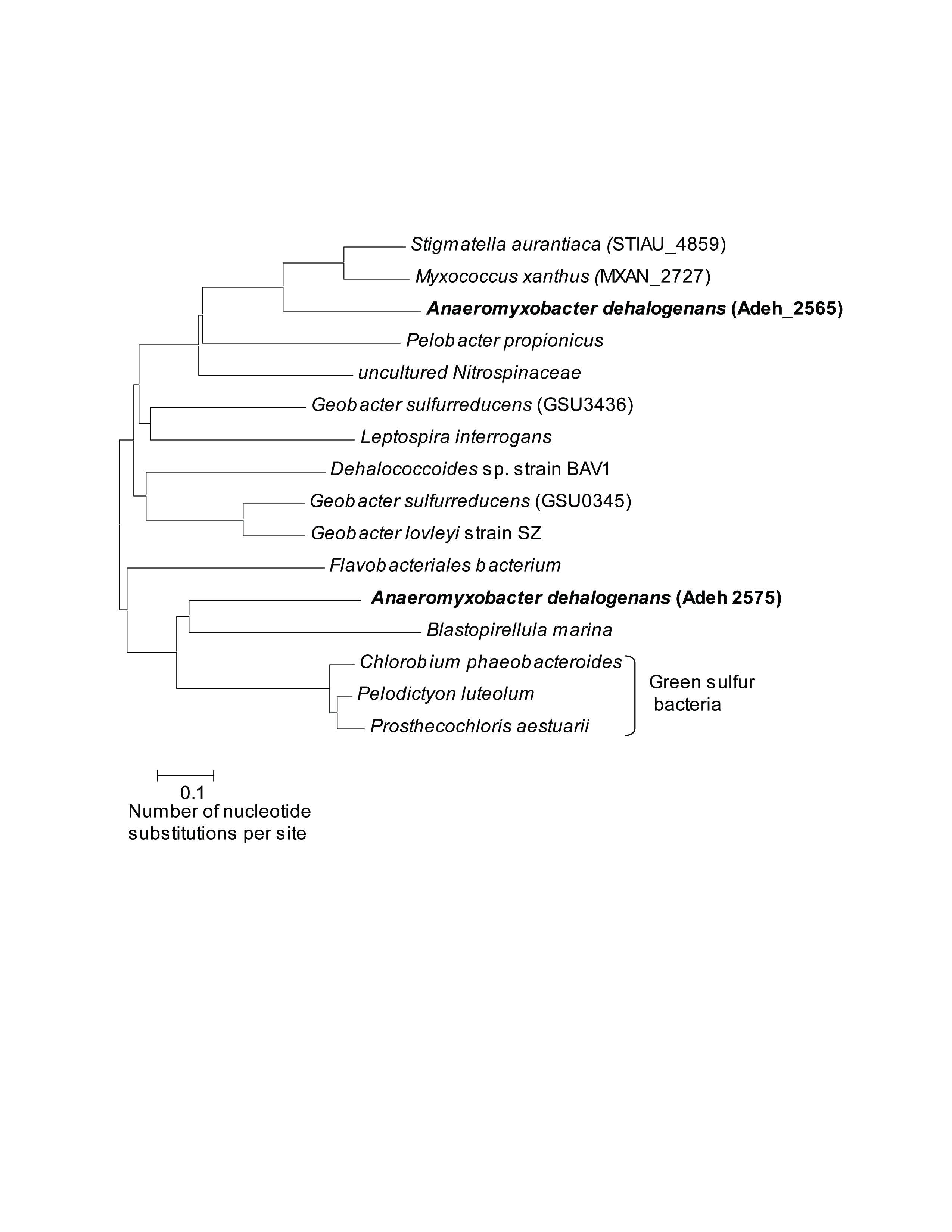

Supplement: Figure S6 — Multiple sequence alignment of A. dehalogenans strain 2CP-C NADH dehydrogenase subunit 1 genes (nuoH) indicates aerobic and anaerobic ancestry. Alignment was made with full-length genes (NCBI database). Locus ID tags for select organisms are indicated in parentheses. (2.87 MB TIF) [file pone.0002103.s006.tif]

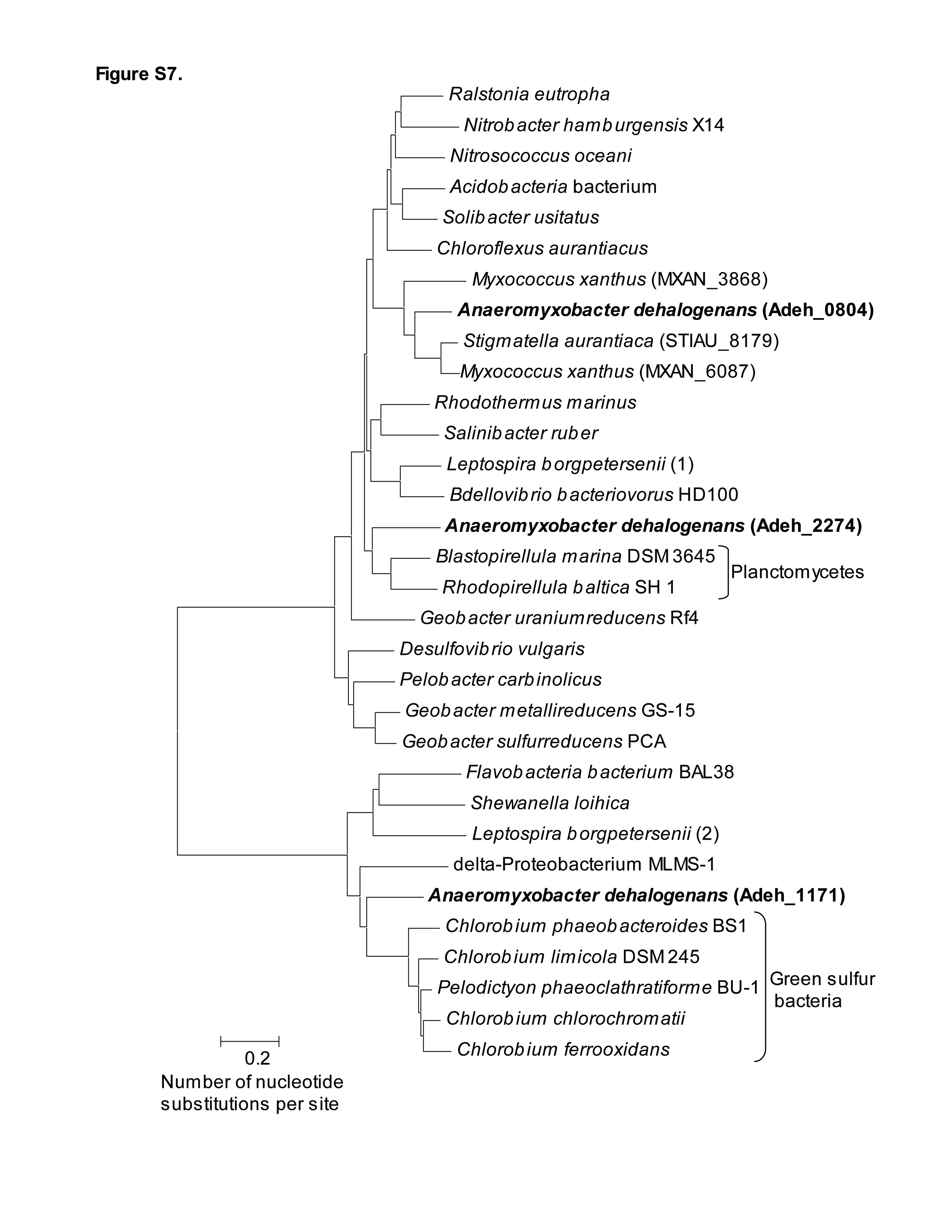

Supplement: Figure S7 — Multiple sequence alignment of A. dehalogenans strain 2CP-C cytochrome oxidase subunit I genes (ctaD or fixN) indicates aerobic and anaerobic ancestry. Alignment was made with full-length genes from the NCBI database. Locus ID tags for select organisms are indicated. (3.91 MB TIF) [file pone.0002103.s007.tif]

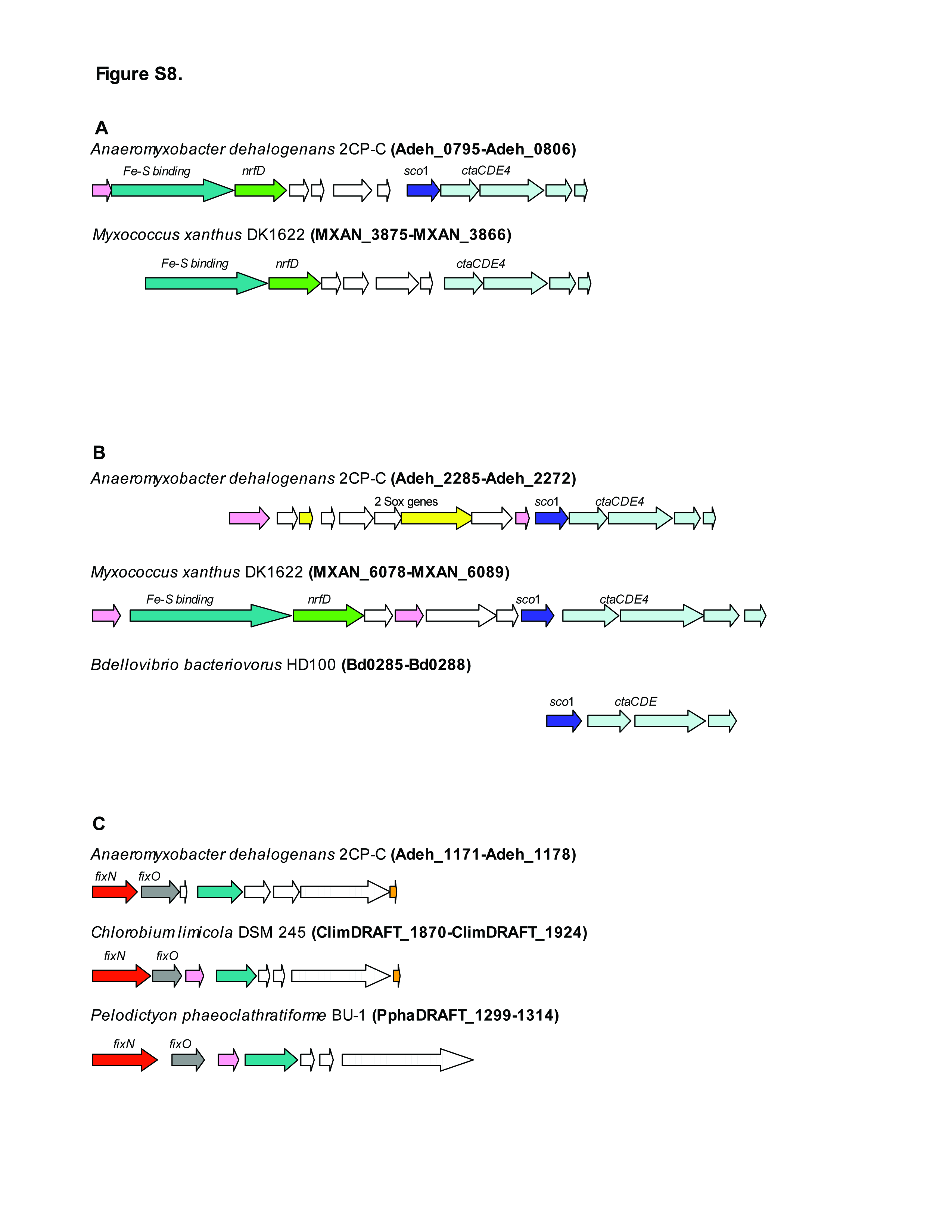

Supplement: Figure S8 — Gene order of cytochrome oxidase gene clusters of A. dehalogenans strain 2CP-C indicate diverse ancestry. Color-coding of arrows indicates clusters with similar gene order and genes with sequence similarity. Colors represent gene annotations as follows: teal, Fe-S binding motif-containing genes; green, polysulphide reductase genes (nrfD); dark blue, synthesis of cytochrome c oxidase genes (sco1); light blue, cytochrome c oxidase subunit genes; pink, c-type cytochrome genes; yellow, sox genes; red-cytochrome cbb3 oxidase subunit 1 genes (fixN); solid grey, cytochrome cbb3 oxidase mono-heme subunit genes (fixO); striped grey, copper-translocating P-type ATPase genes; Orange, cytochrome cbb3 oxidase maturation genes. Hypothetical or non-conserved genes are indicated in white. Locus ID tags are given in parentheses. (A and B) Cytochrome c oxidase clusters syntenous with aerobic organisms. (C) Cytochrome cbb3 oxidase clusters syntenous with anaerobic organisms. (3.05 MB TIF) [file pone.0002103.s008.tif]
