# Supplementary material for: The Mosaic Genome of Anaeromyxobacter dehalogenans Strain 2CP-C Suggests an Aerobic Common Ancestor to the Delta-Proteobacteria
Source: PLoS One. 2008 May 7;3(5):e2103. doi: 10.1371/journal.pone.0002103 (PMC2330069; doi:10.1371/journal.pone.0002103)
Supplement: Table S1 — Anaeromyxobacter dehalogenans strain 2CP-C genome summary. Values indicated are based on both analyses from this study as well as automated annotation reflected in the NCBI genome database. (0.04 MB DOC) [file pone.0002103.s009.doc]

**Table S1.** *Anaeromyxobacter dehalogenans* strain 2CP-C genome summary. Values indicated are based on both analyses from this study as well as automated annotation reflected in the NCBI genome database.

| **Species** | ***Anaeromyxobacter dehalogenans*** |
| --- | --- |
| Strain | 2CP-C |
| Taxonomic position | Myxococcales |
| Accession number | CP000251 |
| Size, bp | 5,013,479 |
| G+C content | 74.91% |
| G+C content in coding areas | 74.82% |
| G+C content in areas outside putative phage regions | 75.21% |
| Predicted number of open reading frames | 4,346 |
| Hypothetical proteins | 1,236 |
| CDS* with top BLAST hits to *Myxococcus xanthus* | 1,057 (24.3%) |
| CDS with top BLAST hits to *Stigmatella aurantiaca* | 737 (17.9%) |
| CDS with top BLAST hits to Myxobacteria | 1,794 (41.2%) |
| CDS with top BLAST hits to *Geobacter* spp. | 282 (6.4%) |
| CDS with top BLAST hits in *Acidobacteria* spp. | 75 (1.7%) |
| Average CDS length | 1050 |
| % of genome coding | 91 |
| rRNA operons | 2 |
| rRNA genes | 6 |
| tRNA genes | 49 |
| Structural RNAs | 58 |
| Regions of deviating G+C content | 15 |
| Phage-related regions | 10 |
| rRNA or tRNA-coding regions | 3 |
| Low G+C regions of unknown origin | 2 |

* Coding sequences (CDS)
