# Supplementary material for: The Mosaic Genome of Anaeromyxobacter dehalogenans Strain 2CP-C Suggests an Aerobic Common Ancestor to the Delta-Proteobacteria
Source: PLoS One. 2008 May 7;3(5):e2103. doi: 10.1371/journal.pone.0002103 (PMC2330069; doi:10.1371/journal.pone.0002103)
Supplement: Table S2 — Putative Horizontal Gene Transfer (HGT) regions based on deviating G+C content and Minimum Codon Adaptation Index (MCAI) HGT calculation was based on phylogenetic origin of lower G+C regions and regions of low codon adaptation index in the A. dehalogenans strain 2CP-C genome. Ten out of 15 G+C regions below 70% contain genes with sequence similarities to phage- or transposon-related genes with E values less than 0.01. Four additional putative HGT regions were identified by codon adaptation index. The genome average MCAI is 0.729. (0.06 MB DOC) [file pone.0002103.s010.doc]

**Table S2.** Putative Horizontal Gene Transfer (HGT) regions based on deviating G+C content and Minimum Codon Adaptation Index (MCAI)

HGT calculation was based on phylogenetic origin of lower G+C regions and regions of low codon adaptation index in the *A. dehalogenans* strain 2CP-C genome. Ten out of 15 G+C regions below 70% contain genes with sequence similarities to phage- or transposon-related genes with *E* values less than 0.01. Four additional putative HGT regions were identified by codon adaptation index. The genome average MCAI is 0.729.

| **Approximate position**  **(bp included in total HGT)** | **G+C %** | **MCAI** | **BLASTp hits to phage-related genes** | ***E* value** |
| --- | --- | --- | --- | --- |
| 651,423-657,132  (5,709 bp) | 61.8 | 0.283 Adeh_0563 | None, tRNA-Asp and tRNA-Val,  adjacent to Adeh_0567 , helicase, prophage LambdaMc01 | e-04 |
| 1,140,945-1,146,257  (5,310 bp) | 75.2 | 0.698 Adeh_0991 | Adeh_0992, *Cytophaga hutchinsonii* | e-30 |
| 1,193,693-1,196,312  (2,619 bp) | 73.3 | 0.362 Adeh_1037 | Adeh_1039, DNA polymerase III subunits gamma and tau, *Bordetella pertussis* (the only gene in this region with prokaryotic hits). | e-06 |
| 1,619,133-1,625,971  (6,838 bp) | 65.4 | 0.253 Adeh_1408 | Adeh_1411, transposase, *Marinobacter algicola* | e-91 |
| 1,684,675-1,687,653  (2,978 bp) | 69.7 | 0.312 Adeh_1463 | Adeh_1462, integrase, *Stigmatella aurantiaca*  *Lactobacillus johnsonii* prophage  Adeh_1463, excisionase, *Plesiocystis pacifica* SIR-1  Mycobacteriophage Che12  Adeh_1467, resolvase-like protein, *Ralstonia solanacearum* phage RSM1 | e-67  e-10  0.86  e-25 |
| 1,813070-1,829,454  Not included in total | 67.0 | 0.673 | None, tRNA-Thr, tRNA-Tyr, tRNA-Gly, tRNA-Thr, and tRNA-Trp |  |
| 1,997,218-200,1552  Not included in total | 68.1 | 0.552 | None, Adeh_1743, LysR regulator, Myxococcaceae | <e-26 |
| 2,034,615-2,058,673 (24,058 bp) | 74.9 | 0.228 Adeh_1794 | Adeh_1801, terminase small subunit, Enterobacteria phage epsilon15 | e-07 |
| 2,097,739-2,111,883 (14,144 bp) | 66.5 | 0.263 Adeh_1845 | Adeh_1835, integrase, *Myxococcus xanthus*  Mycobacteriophage CJW1  Adeh_1839, putative integrase, *Pseudomonas* phage M6 | e-10  e-04  e-60 |
| 2,128,510-2,143,067 (14,557 bp) | 70.5 | 0.356 Adeh_1876 | Adeh_1877, *Myxococcus* phage Mx8 | e-10 |
| 2,168,208-2,174,240  Not included in total | 69.5 | 0.690 | None, ribosomal genes. |  |
| 2,177,427-2,204,940  Not included in total | 69.5 | 0.689 | None, ribosomal genes. |  |
| 2,619,127-2,632,994 (13,867 bp) | 63.5 | 0.345 Adeh_2326 | Adeh_2331, Cin, *Enterobacteria* phage P1 virion  Adeh_2336, recombinase, Bacteriophage phBC6A51  Adeh_2327, HsdR site-specific deoxyribonuclease, *Chlorobium phaeobacteroides* DSM 266  Adeh_2328, type I restriction, *Methanosarcina barkeri* str. Fusaro | e-06  e-17  0  e-78 |
| 3,244,090-3,251,195  (7,105 bp) | 69.2 | 0.436 Adeh_2868 | Adeh_2871, death-on-curing protein sequence, *Candidatus Protochlamydia* *amoepophila* bacteriophage P1 | e-16 |
| 3,661,055-3,733,532 (72,477 bp) | 67.0 | 0.210 Adeh_3214 | Adeh_3209, XRE family regulator, *Candidatus Protochlamydia amoebophila* UWE25  Adeh_3213, putative transposase, *Rhodococcus* sp. RHA1  Adeh_3219, LysR regulator, has top blastp hits within Myxococcaceae | e-9  e-26  <e-58 |
| 3,962,432-3,956,308  (6,124 bp) | 67.5 | 0.269 Adeh_3442 | Adeh_3442, DNA-cytosine methyltransferase, gp127 Mycobacterium phage Omega | e-6 |
| 4,064,430-4,090,399 (26,079 bp) | 65.4 | 0.263 Adeh_3537 | Adeh_3551, hypothetical protein, Mycobacteriophage Cooper | e-10 |
| 4,143,301-4,164,184 (20,883 bp) | 69.9 | 0.231  Adeh_3618 | Adeh_3617, hypothetical, Mycobacteriophage CJW1  Adeh_3618, resolvase, *Methylobacterium extorquens*  *Geobacillus* virus E2  Adeh_3627, HNH endonuclease, *Solibacter usitatus*  gp30 in *Streptomyces* phage phiC31 | e-81  e-14  e-06  e-15 0.12 |
| 4,437,523-4,447,517  (9,994 bp) | 68.7 | 0.341  Adeh_3864 | Adeh_3869, helicase, *Lactobacillus acidophilus* NCFM | e-62 |
| 4,917,551-4,928,537 (10,986 bp) | 62.4 | 0.205 Adeh_4278 | None. Adjacent to Adeh_4278, GDP-mannose 4,6-dehydratase, Cyanophage P-SSM2 | e-92 |
| **Total HGT=243,728 bp** |  |  |  |  |
