# Supplementary material for: The Mosaic Genome of Anaeromyxobacter dehalogenans Strain 2CP-C Suggests an Aerobic Common Ancestor to the Delta-Proteobacteria
Source: PLoS One. 2008 May 7;3(5):e2103. doi: 10.1371/journal.pone.0002103 (PMC2330069; doi:10.1371/journal.pone.0002103)
Supplement: Table S3 — Genes for adventurous motility proteins on the A. dehalogenans strain 2CP-C genome imply that this type of motility is present. E values and identities given refer to M. xanthus sequences. Many of the gliding motility genes identified in M. xanthus are present in A. dehalogenans strain 2CP-C but whether or not the genes present are sufficient to produce gliding motility is unknown. (0.05 MB DOC) [file pone.0002103.s011.doc]

**Table S3.** Genes for adventurous motility proteins on the *A. dehalogenans* strain 2CP-C genome imply that this type of motility is present. *E* values and identities given refer to *M. xanthus* sequences. Many of the gliding motility genes identified in *M. xanthus* are present in *A. dehalogenans* strain 2CP-C but whether or not the genes present are sufficient to produce gliding motility is unknown.

| **Protein name** | ***E* value** | **Identity*** |
| --- | --- | --- |
| AglR | e-76 | 147/244 (60%) |
| AglS | e-34 | 76/168 (45%) |
| AglT | e-68 | 166/460 (66%) |
| AglU | e-47 | 171/536 (31%) |
| AglW | e-107 | 202/430 (46%) |
| AglX | e-72 | 135/238 (56%) |
| AglZ* | e-41 | 219/583 (37%) |
| AgmA | e-66 | 191/578 (33%) |
| AgmB | Sequence not available | |
| AgmC | e-37 | 90/185 (48%) |
| AgmD | e-48 | 125/346 (36%) |
| AgmE | e-108 | 196/315 (62%) |
| AgmF | e-78 | 155/262 (59%) |
| AgmG | No similar *A. dehalogenans* protein | |
| AgmH | e-60 | 123/277 (43%) |
| AgmI | e-37 | 112/288 (38%) |
| AgmJ | No similar *A. dehalogenans* protein | |
| AgmK | 0 | 1119/2521 (44%) |
| AgmL | 0 | 327/429 (76%) |
| AgmM | e-23 | 88/298 (29%) |
| AgmN | No similar *A. dehalogenans* protein | |
| AgmO | No similar *A. dehalogenans* protein | |
| AgmP | e-48 | 122/307 (39%) |
| AgmQ | No similar *A. dehalogenans* protein | |
| ArmR | e-96 | 191/370 (51%) |
| AgmS | e-37 | 101/250 (40%) |
| AgmT | e-18 | 83/304 (27%) |
| AgmU | 0 | 474/1231 (38%) |
| AgmV | e-13 | 227/796 (28%) |
| AgmW | e-102 | 205/436 (47%) |
| AgmX | e-76 | 215/683 (31%) |
| AgmY | Sequence not available | |
| AgmZ | e-8 | 42/129 (32%) |
| CglB | No similar *A. dehalogenans* protein | |
| MglA | e-97 | 171/195 (87%) |

* Percent identities represent the number of amino acids of the *A. dehalogenans* translated protein that are common with its *M. xanthus* protein counterpart divided by the total number of amino acids in the comparison.
