# Supplementary material for: The Mosaic Genome of Anaeromyxobacter dehalogenans Strain 2CP-C Suggests an Aerobic Common Ancestor to the Delta-Proteobacteria
Source: PLoS One. 2008 May 7;3(5):e2103. doi: 10.1371/journal.pone.0002103 (PMC2330069; doi:10.1371/journal.pone.0002103)
Supplement: Table S4 — The A. dehalogenans strain 2CP-C genome contains multiple copies of protease and chaperone genes. The E values and identities refer to a comparison with M. xanthus genes. Of the 19 genes identified encoding proteolytic enyzmes in the strain 2CP-C genome, 14 have homologs in M. xanthus. (0.04 MB DOC) [file pone.0002103.s012.doc]

**Table S4.** The *A. dehalogenans* strain 2CP-C genome contains multiple copies of protease and chaperone genes. The *E* values and identities refer to a comparison with *M. xanthus* genes. Of the 19 genes identified encoding proteolytic enyzmes in the strain 2CP-C genome, 14 have homologs in *M. xanthus*.

| Protein name | *E* value | Identity* |
| --- | --- | --- |
| LonB1 | No *M. xanthus* homolog | |
| Lon2 | 0 | 674/803 (83%) |
| LonC3 | No *M. xanthus* homolog | |
| Lon4 | e-24 | 73/157 (46%) |
| FtsH1 | 0 | 423/634 (66%) |
| FtsH2 | 0 | 416/606 (68%) |
| FtsH3 | *S. aurantiaca* homolog only (e-147) | |
| DnaJ1 | e-38 | 93/218 (42%) |
| DnaJ2 | No *M. xanthus* homolog | |
| DnaJ3 | e-73 | 213/512 (41%) |
| DnaJ4 | No *M. xanthus* homolog | |
| DnaJ5 | e-14 | 42/68 (61%) |
| DnaJ6 | e-37 | 92/176 (52%) |
| DnaJ7 | No *M. xanthus* homolog | |
| DnaJ8 | e-113 | 215/370 (58%) |
| ClpX1 | 0 | 344/424 (81%) |
| ClpX2 | 0 | 332/415 (80%) |
| DnaK1 | 0 | 484/635 (76%) |
| DnaK2 | e-153 | 473/938 (50%) |
| DnaK3 | 0 | 474/606 (78%) |

* Percent identities represent the number of amino acids of the *A. dehalogenans* translated protein that are common with its *M. xanthus* protein counterpart divided by the total number of amino acids in the comparison.
