# Supplementary material for: The Mosaic Genome of Anaeromyxobacter dehalogenans Strain 2CP-C Suggests an Aerobic Common Ancestor to the Delta-Proteobacteria
Source: PLoS One. 2008 May 7;3(5):e2103. doi: 10.1371/journal.pone.0002103 (PMC2330069; doi:10.1371/journal.pone.0002103)
Supplement: Table S5 — Flagellar motility genes on the A. dehalogenans strain 2CP-C genome. According to the currently accepted Salmonella and E. coli models, almost all the genes necessary for flagellum synthesis and export are present in a coherent cluster on the A. dehalogenans genome with the exception of four chaperone genes (fliJ, flgN, fliT, flgA), a rod capping protein flgJ, and a hook-length-control protein fliK. (0.05 MB DOC) [file pone.0002103.s013.doc]

**Table S5.** Flagellar motility genes on the *A. dehalogenans* strain 2CP-C genome. According to the currently accepted *Salmonella* and *E. coli* models, almost all the genes necessary for flagellum synthesis and export are present in a coherent cluster on the *A. dehalogenans* genome with the exception of four chaperone genes (*fliJ*, *flgN*, *fliT*, *flgA*), a rod capping protein *flgJ*, and a hook-length-control protein *fliK*.

| Gene name | Locus ID | Closest homolog | *E* value | Identity* |
| --- | --- | --- | --- | --- |
| *fliA* | Adeh_1355 | *Chromohalobacter salexigens* | e-21 | 89/197 (45%) |
| *fliC* | Adeh_1339; Adeh_1342 | *Bdellovibrio bacteriovorus* | e-46 | 135/254 (53%) |
| *fliD* | Adeh_1341 | *Geobacter sulfurreducens* | e-35 | 144/465 (30%) |
| *fliE* | Adeh_1395 | *Syntrophus aciditrophicus* | e-5 | 21/39 (53%) |
| *fliF* | Adeh_1394 | *Geobacter* sp. FRC-32 | e-46 | 149/375 (39%) |
| *fliG* | Adeh_1393 | *Thermotoga maritima* | e-32 | 109/312 (34%) |
| *fliH* | Adeh_1392 | *Blastopirellula marina* | e-4 | 30/100 (30%) |
| *fliI* | Adeh_1391 | *Desulfitobacterium hafniense* | e-90 | 217/401 (54%) |
| *fliL* | Adeh_1366 | *Lawsonia intracellularis* | e-7 | 32/96 (33%) |
| *fliM* | Adeh_1365 | *Nitrosospira multiformis* | e-20 | 79/259 (30%) |
| *fliN* | Adeh_1364 | *Lawsonia intracellularis* | e-26 | 61/94 (64%) |
| *fliO* | Adeh_1363 | *Geobacter metallireducens* | 0.002 | 37/106 (34%) |
| *fliP* | Adeh_1362 | delta-Proteobacterium MLMS-1 | e-47 | 113/217 (52%) |
| *fliQ* | Adeh_1361 | *Aquifex aeolicus* | 0.35 | 16/38 (42%) |
| *fliR* | Adeh_1360 | *Saccharophagus degradans* | e-15 | 62/220 (28%) |
| *fliS* | Adeh_1338 | *Bdellovibrio bacteriovorus* | 0.91 | 24/84 (28%) |
| *flbD* | Adeh_1386 | *Desulfitobacterium hafniense* | 0.001 | 21/35 (60%) |
| *motA* | Adeh_1385 | *Chromobacterium violaceum* | e-54 | 112/222 (50%) |
| *motB* | Adeh_1384 | *Geobacter sulfurreducens* | e-30 | 82/242 (33%) |
| *flhA* | Adeh_1358 | *Pelobacter carbinolicus* | e-102 | 282/630 (44%) |
| *flhB* | Adeh_1359 | *Ralstonia solanacearum* | e-28 | 70/135 (51%) |
| *flhF* | Adeh_1357 | *Clostridium tetani* | e-31 | 73/196 (37%) |
| *flhG* | Adeh_1356 | *Pelobacter carbinolicus* | e-54 | 124/291 (42%) |
| *flgB* | Adeh_1397 | marine gamma-Proteobacterium | e-4 | 22/44 (50%) |
| *flgC* | Adeh_1396 | *Desulfovibrio vulgaris* | e-28 | 68/145 (46%) |
| *flgD* | Adeh_1388 | *Geobacter metallireducens* | e-31 | 78/192 (40%) |
| *flgE* | Adeh_1387 | *Geobacter metallireducens* | e-61 | 185/422 (43%) |
| *flgF* | Adeh_1352 | *Acidovorax avenae subsp. citrulli* | e-19 | 81/237 (34%) |
| *flgG* | Adeh_1351 | *Halothermothrix orenii* | e-59 | 123/258 (47%) |
| *flgH* | Adeh_1349 | *Syntrophus aciditrophicus* | e-30 | 71/185 (38%) |
| *flgI* | Adeh_1348 | *Rhodopseudomonas palustris* | e-62 | 169/343 (49%) |
| *flgK* | Adeh_1344 | *Geobacter metallireducens* | e-19 | 138/454 (30%) |
| *flgL* | Adeh_1343 | *Geobacter sulfurreducens* | e-13 | 54/169 (31%) |

* Percent identities represent the number of amino acids of the *A. dehalogenans* translated protein that are common with its closest homolog divided by the total number of amino acids in the comparison.
