# Supplementary material for: The Mosaic Genome of Anaeromyxobacter dehalogenans Strain 2CP-C Suggests an Aerobic Common Ancestor to the Delta-Proteobacteria
Source: PLoS One. 2008 May 7;3(5):e2103. doi: 10.1371/journal.pone.0002103 (PMC2330069; doi:10.1371/journal.pone.0002103)
Supplement: Table S6 — Reactive Oxygen Species (ROS)-detoxification gene comparison across selected delta-proteobacteria genomes (Myxococcus xanthus DK1622, Anaeromyxobacter dehalogenans 2CP-C, Geobacter sulfurreducens PCA, and Desulfovibrio vulgaris Hildenborough) indicates that the A. dehalogenans strain 2CP-C genome combines aerobic with anaerobic strategies for detoxifying ROS. (0.04 MB DOC) [file pone.0002103.s014.doc]

**Table S6.** Reactive Oxygen Species (ROS)-detoxification gene comparison across selected delta-proteobacteria genomes (*Myxococcus xanthus* DK1622, *Anaeromyxobacter dehalogenans* 2CP-C, *Geobacter sulfurreducens* PCA, and *Desulfovibrio vulgaris* Hildenborough) indicates that the *A. dehalogenans* strain 2CP-C genome combines aerobic with anaerobic strategies for detoxifying ROS.

| **Enzyme** | **Reaction catalyzed** | **Locus ID tags** | | | |
| --- | --- | --- | --- | --- | --- |
|  |  | *M. xanthus* | *A. dehalogenans* | *G. sulfurreducens* | *D. vulgaris* |
| Catalases | H_2_O_2_ + H_2_O_2_ → 2 H_2_O + O_2_ | MXAN_6188 MXAN_4389 | Absent | GSU2100 | DVUA0091 |
| Superoxide dismutases | O_2_^-^ + O_2_^-^ + 2H^+^ → H_2_O_2_ + O_2_ | MXAN_5862 MXAN_4826 | Adeh_1952 | GSU1158 | DVU2410 |
| Superoxide reductases | O_2_^-^ + 2H^+^ + cyt c_reduced_ → H_2_O_2_ + cyt c_oxidized_ | Absent | Absent | Absent | DVU3183 |
| Alkylhydro-peroxidases | H_2_O_2_ + NADH + H^+^ → 2 H_2_O + NAD^+^ | MXAN_1564 MXAN_1563 MXAN_5223 | Adeh_0313 Adeh_0314 Adeh_3128 | GSU3246  GSU0893 | Absent |
| Thiol peroxidase | H_2_O_2_ + NADH + H^+^ → 2 H_2_O + NAD^+^ | MXAN_6496 | Adeh_1146 Adeh_1148 Adeh_0828 | GSU0352 | DVU1228 |
| Cytochrome c peroxidases | H_2_O_2_ + NADH + H^+^ → 2 H_2_O + NAD^+^ | MXAN_0977 MXAN_7213 MXAN_5562 MXAN_5801 | Absent | GSU2813 GSU0466 | Absent |
| Rubrerythrin | H_2_O_2_ + NADH + H^+^ → 2 H_2_O + NAD^+^ | Absent | Adeh_0765 Adeh_2075 Adeh_3092* Adeh_2193* | GSU2814* GSU2612* | DVU3094  DVU2310  DVU0019 |
| Rubredoxin | Polyglucose^†^ + NADH + H^+^ → glucose^†^ + NAD^+^ | Absent | Adeh_3092* Adeh_2193* | GSU2814*  GSU2612*  GSU0847  GSU3188 | DVU3184 |
| Rubredoxin:oxygen oxidoreductase | O_2_ + 4H^+^ → 2H_2_O NO → N_2_O | Absent | Absent | GSU3294 | DVU3185 |

*GSU2814, GSU2612, Adeh_3092, and Adeh_2193 encode rubrerythrin:rubredoxin fused proteins

^†^Polyglucose is the electron donor in the rubredoxin reaction of *D. vulgaris*; the donor is unknown in other organisms
